# BET inhibition as a new strategy for the treatment of gastric cancer

**DOI:** 10.18632/oncotarget.9766

**Published:** 2016-06-01

**Authors:** Raquel C. Montenegro, Peter G.K. Clark, Alison Howarth, Xiao Wan, Alessandro Ceroni, Paulina Siejka, Graciela A. Nunez-Alonso, Octovia Monteiro, Catherine Rogers, Vicki Gamble, Rommel Burbano, Paul E. Brennan, Cynthia Tallant, Daniel Ebner, Oleg Fedorov, Eric O'Neill, Stefan Knapp, Darren Dixon, Susanne Müller

**Affiliations:** ^1^ The Structural Genomics Consortium, Nuffield Department of Medicine, University of Oxford, Headington, Oxford OX3 7DQ, UK; ^2^ Target Discovery Institute, Nuffield Department of Medicine, University of Oxford, Headington, Oxford OX3 7FZ, UK; ^3^ Department of Chemistry, Chemistry Research Laboratory, University of Oxford, Oxford OX1 3TA, UK; ^4^ Federal University of Pará, Institute of Biological Sciences, Belém, Pará 66075-110, Brazil; ^5^ Institute for Pharmaceutical Chemistry and Buchmann Institute for Life Sciences, Frankfurt am Main D-60438, Germany; ^6^ CRUK/MRC Oxford Institute of Radiation Biology, University of Oxford, Headington OX3 7DQ, UK

**Keywords:** epigenetic, bromodomain, BET inhibitors, cytotoxicity, gastric cancer

## Abstract

Gastric cancer is one of the most common malignancies and a leading cause of cancer death worldwide. The prognosis of stomach cancer is generally poor as this cancer is not very sensitive to commonly used chemotherapies. Epigenetic modifications play a key role in gastric cancer and contribute to the development and progression of this malignancy. In order to explore new treatment options in this target area we have screened a library of epigenetic inhibitors against gastric cancer cell lines and identified inhibitors for the BET family of bromodomains as potent inhibitors of gastric cancer cell proliferations. Here we show that both the pan-BET inhibitor (+)-JQ1 as well as a newly developed specific isoxazole inhibitor, PNZ5, showed potent inhibition of gastric cancer cell growth. Intriguingly, we found differences in the antiproliferative response between gastric cancer cells tested derived from Brazilian patients as compared to those from Asian patients, the latter being largely resistant to BET inhibition. As BET inhibitors are entering clinical trials these findings provide the first starting point for future therapies targeting gastric cancer.

## INTRODUCTION

The global burden of cancer continues to increase largely because of an aging population and an increasing adoption of cancer-causing life style behaviours [1]. Among the different kinds of cancer, gastric cancer (GC) is one of the most common malignancies, remaining a major public health issue as the fifth most common cancer and the second leading cause of cancer death worldwide [2, 3]. Gastric cancer is an aggressive disease, and its prognosis remains poor, generally owing to the absence of specific symptoms that renders early diagnosis of this disease difficult [4]. About half of GC patients have recurrent disease after curative surgery, but the outcome for these patients is still very poor, with an estimated 5-year survival rate for locoregional disease of 25% - 35% [5, 6]. Although various treatment modalities have been developed, many of them have failed to eliminate GC cells curatively [7]. The current standard of care for metastatic GC patients is palliative chemotherapy providing the best supportive care, aimed at extending survival of the patients from 3 months with no therapy to 7–15 months with modern combination chemotherapy, and a dismal 2% 5-year survival rate with chemotherapy [8]. Clinical trials evaluating novel targeted therapies identified HER-2 as a target for GC. A recent ‘Trastuzumab for Gastric Cancer’ (ToGA) trial successfully demonstrated trastuzumab (Trastuzumab^®^) a recombinant humanized monoclonal antibody that targets the extracellular domain IV of the HER2 protein, as the first biologic therapy to have activity in advanced GC. However, only 10–25% of GC samples showed HER-2 amplification and even in those cases the ToGA study showed an improvement of a significant, but poor median survival by 2.5 months for patients treated with classical chemotherapy plus trastuzumab [9]. Therefore, there is an urgent need to introduce new therapeutic agents for the clinical management of gastric cancer.

Epigenetic modifications play a key role in several types of cancer, including gastric cancer [10, 11], which ultimately contributes to the development and progression of this malignancy [12, 13]. The stomach has been described as one of the organs with increased frequency of hypermethylation of CpG islands, which has been associated with age and inflammatory processes [14], potentially related to the exposure of the tissue to exogenous agents. Targeting of epigenetic processes may therefore provide a new method in the treatment of gastric cancer.

Bromodomains (BRDs) are conserved chromatin binding domains found in many nuclear proteins. BRD containing proteins are specifically recruited to ε-N-acetylated lysine residues present in histones and other proteins. BRD containing proteins function as regulators of gene transcription and by modulating and modifying chromatin structure [15–18]. The dysfunction of BRD containing proteins has been linked to the development of various types of cancer [19]. In particular, proteins of the bromo and extra terminal (BET) family, namely BRD2, BRD3, BRD4 and BRDT, have been identified as regulators for the expression of key oncogenes and the first clinical trials using BET inhibitors have recently commenced [15, 18, 20–24]. However, also the first resistance mechanisms have been reported [25, 26].

**Figure 1:**
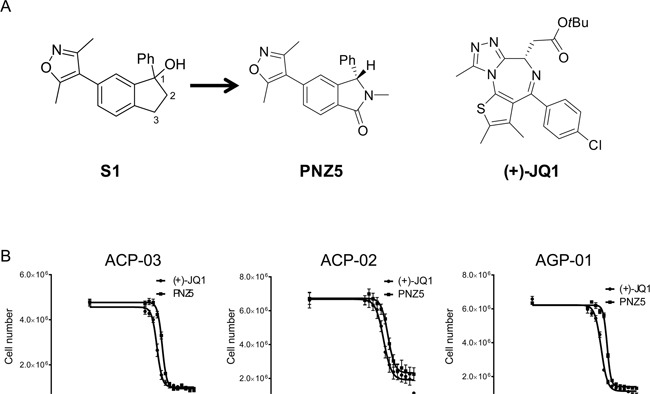
**A.** BET inhibitors used in the study (+)-JQ1 and (PNZ5) as well as lead compound S1 **B.** Growth inhibition curves of three gastric cancer cell lines derived from Brazilian patients after treatment with BET inhibitors (+)-JQ1 and PNZ5 for 72h.

Given the dire need for anticancer agents active against gastric cancer, the effect of an epigenetic inhibitor library on GC cells was assessed in both 2D and 3D *in vitro* models. BET family inhibitors (Figure 1A) were identified as the first potent epigenetic inhibitors of gastric cancer cells. Both the pan-BET inhibitor (+)-JQ1 as well as a newly developed isoxazole, PNZ5, showed potent inhibition of GC cells providing a starting point for future therapy (Figure 1B).

## RESULTS

### Profiling of epigenetic probes in gastric cancer cell lines

Initial screening with a small library of 19 epigenetic probes was performed in three GC cell lines, but only 5 compounds (Bromosporine, UN1999, UNC0638, (+)-JQ1 and PNZ5) inhibited the growth of the cells at a concentration of 10 μM after 72h incubation (Supplementary Table 1). (+)-JQ1 and PNZ5 were the most active compounds, with strong antiproliferative activity and were therefore chosen for further investigation (Figure 1B).

### PNZ5 is a potent pan-BET inhibitor

Isoxazoles have been explored previously as BET bromodomain inhibitors [27–29]. We have developed PNZ5, a new isoxazole-based inhibitor originating from the lead compound S1 (Figure 1). PNZ5 was developed as the result of a structure-based lead optimisation program. Dihydroindene S1 was identified as a ligand against BRD4(1) (pIC_50_ 5.9) [30]. Investigation of the co-crystal structure of S1 with BRD4(1) [PDB ID 4GPJ] identified a number of positions for potential optimisation: addition of a carbonyl group at C-3 was envisaged to benefit from hydrogen-bonds (H-bonds) to a network of conserved water molecules; replacement of C-2 with a nitrogen would minimise interactions in the narrow ZA channel of BRD4(1) and *N*-substitution could provide further binding interactions with the protein; and alternative substitution of the hydroxyl at C-1 would provide a handle for further optimisation. Incorporation of a *N*-methyl lactam motif (S2) resulted in a signficant increase in the affinity, validating the new scaffold. Substitution at both C-2 and in place of the C-1 hydroxyl were found to be largely detrimental to affinity for BRD4(1), with proto-substituents in each position optimal for binding. The three best compounds, S3, S5 and S7, were destined to be separated into their component enantiomers for further evaluation (Supplementary Table 2). However, S7 was found to be unstable under the separation conditions and S5 was inseparable with the equipment available to us, but, pleasingly, S3 was readily resolved by preparative chiral HPLC. Biophysical assays on the two enantiomers revealed the *S*-enantiomer, PNZ5, as the biologically active species, which was then used for further biological interrogation of BRD4(1). The synthesis and analytical data are summarized in Scheme S1.

**Figure 2:**
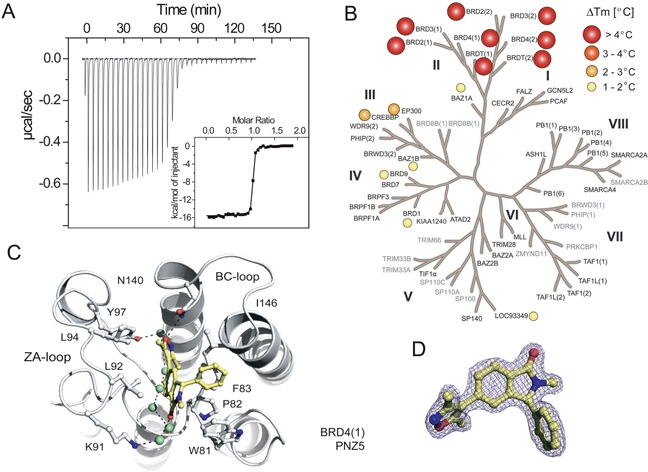
Characterization of PNZ5 **A.** Isothermal titration data of the interaction of PNZ5 with the first bromodomain of BRD4 (BRD4(1)). Shown are the raw binding heats for each injection as well as normalized binding enthalpies. **B.** Selectivity of PNZ5 calculated using thermal shift (ΔTm) assays. Screened targets (labelled in black) in the phylogenetic tree based on the BRD family structure based alignment [71]. **C.** Binding mode of PNZ5 bound to BRD4(1). Hydrogen bonds to the conserved asparagine (N140) and tyrosine (Y97) are shown as dotted green lines. **D.** Electron density map (2FoFc) contoured at 2σ around the inhibitor.

Potency of PNZ5 for the first bromodomain of BRD4(1) was assessed by isothermal titration calorimetry (ITC) revealing a dissociation constant (*K*_D_) of 5.43 nM with a favourable enthalpic contribution of −15.57 kcal/mol (Figure 2A and Supplementary Table 3). The compound is highly selective for the BET family as assessed by a comprehensive thermal shift assay (Figure 2B and Supplementary Table 5). Since its discovery in our own labs, similar inhibitors have also been disclosed by researchers at Boehringer Ingelheim [reference: patent, WO 2014/154760 [31]]. The inhibitor contains two possible acetyl-lysine mimetic moieties. In order to obtain molecular insights on the interaction of the developed inhibitor, the crystal structure of the first bromodomain of BRD4 in complex with PNZ5 was solved at 1.85 Å (Figure 2C and Supplementary Table 4, 5FBX.pdb). The inhibitor, coordinating residues and water molecules were well defined by the electron density. The dimethylisoxazole moiety mimicked the acetyl lysine side chain and formed two canonical hydrogen-bond interactions with Asn140 and water mediated hydrogen-bond with Tyr 97 (Figure 2C). The common network of waters within the binding site was also conserved in the BRD4(1)-PNZ5 complex. The compound was further stabilized by lipophilic interactions with residues forming the binding site Leu92, Leu94, Ile146. The phenyl substituent was well accommodated next to the WPF shelf, as well as the N1-methyl substituent that formed hydrophobic interactions with Trp81. The carbonyl on the isoindolinone established a water-mediated hydrogen bond with Lys91. Superposition of the apo structure of BRD4(1) (2OSS.pdb) with the structure in complex with PNZ5 did not reveal any major rearrangements in the ZA-loop segment.

### (+)-JQ1 and PNZ5 alter intracellular motility of BRD4

**Figure 3:**
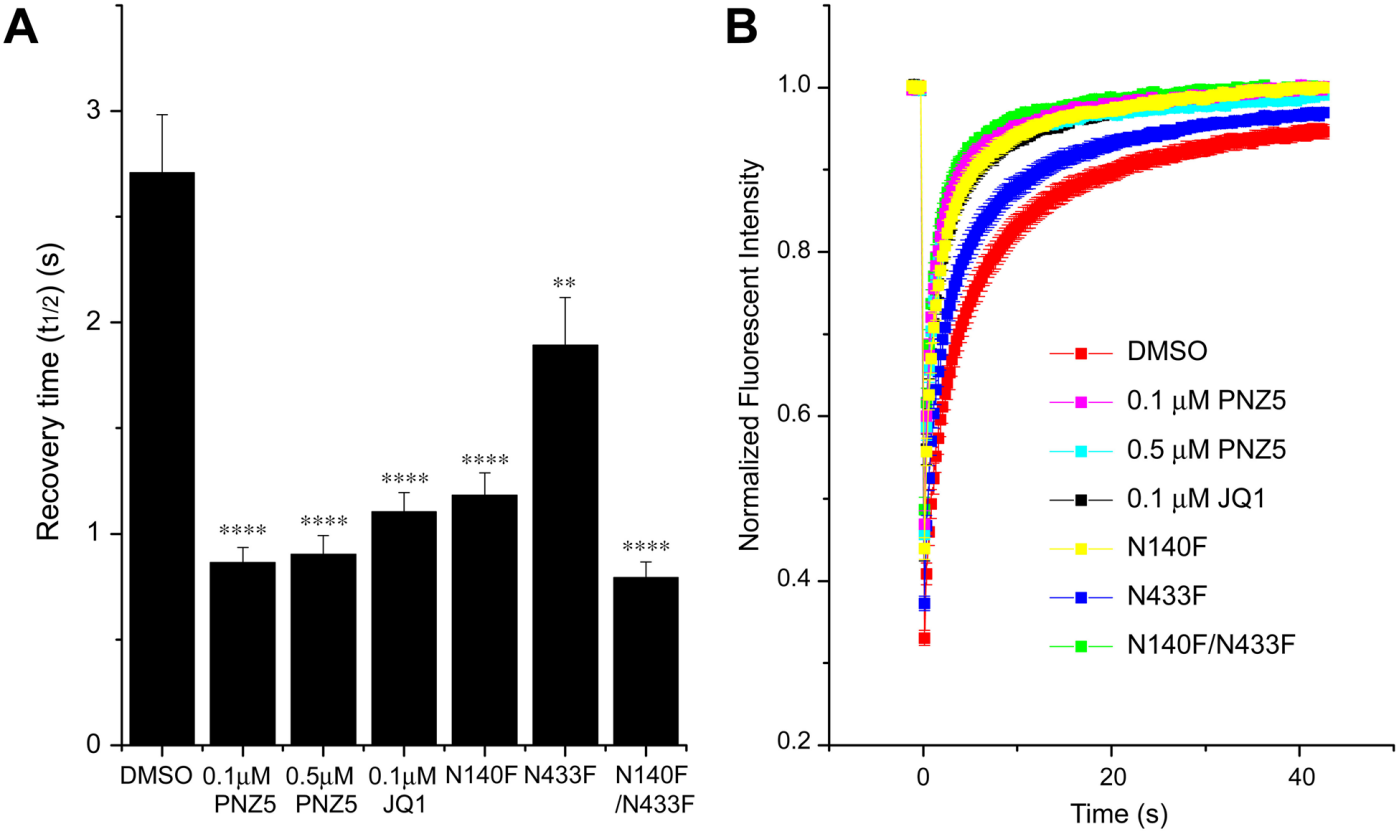
Effect of (+)-JQ1 and PNZ5 on intracellular motility of BRD4 **A.** Fluorescence recovery half-times for full-length BRD4. PNZ5 significantly decreased half recovery times of U2OS cells transfected with wild-type full-length GFP-BRD4, as did (+)-JQ1. Half recovery times were accelerated to the level of the double bromodomain mutant N140F/N1433F and faster than the single mutant. **B.** Time dependence of the fluorescence recovery of the bleached area. At least 20 nuclei were bleached for each experiment and the mean recovery time as well as the standard error of mean (SEM) are shown in A (****p<0.0001; ** p<0.002).

Cellular activity of PNZ5 was demonstrated using a full length BRD4 fluorescence recovery after photobleaching (FRAP) assay [32]. The data showed that at a concentration of ≤0.5 μM, PNZ5 effectively reduced the fluorescent half-time recovery of BRD4 fused to GFP to levels comparable to both the BET inhibitor (+)-JQ1 and the double mutant N140F/N433F, which is unable to bind to chromatin (Figure 3). The effect on fluorescence recovery was stronger than seen for the single domain mutant N140F of the first bromodomain or N433F of the second bromodomain, respectively. These findings suggested that PNZ5 effectively displaced both bromodomains of ectopically expressed BRD4 from chromatin.

### Diverse gastric cancer cell lines differ in *BRD4* and c-*MYC* expression levels

**Figure 4:**
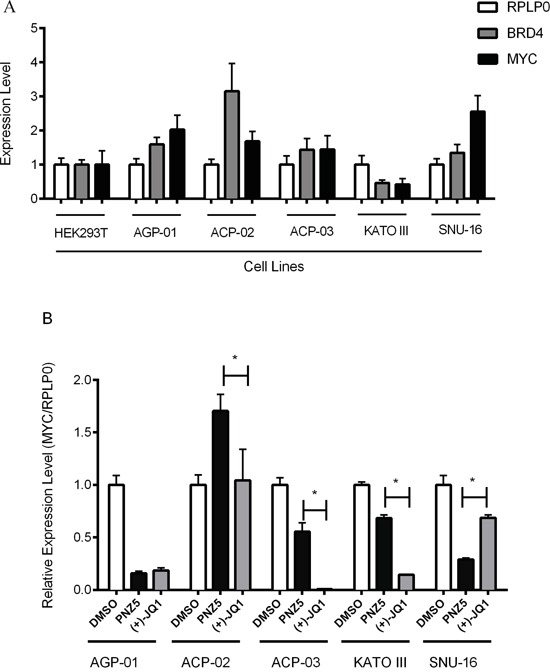
Expression profile of *BRD4* and *c-MYC* mRNA levels by qRT–PCR in a panel of gastric cancer cell lines **A.** Expression of *BRD4* was evaluated in a panel of human gastric cancer cell lines and compared with HEK293T cells. **B.** Expression of *c-MYC* was evaluated in all gastric cell lines after 6 h treatment with (+)-JQ1 and PNZ5 (500 nM). The mRNA level for *BRD4* and *c-MYC* was normalized to *RPLP0* mRNA level in each cell line. Average and standard deviation were calculated from two independent experiments in triplicate.

We aimed to explore if sensitivity to BET inhibitors was a general feature of gastric cancer and assessed the effect of BET inhibition on GC lines of Asian and Brazilian origins. The 2 Asian cell lines are derived from metastatic sites. The Brazilian cell lines represent different tumors: ACP-02, is a diffuse type GC; ACP-03 an intestinal type and AGP-01 represents a malignant ascites. Interestingly, BET inhibitors did not have an effect on proliferation of the 2 cell lines originating from Asian patient cohorts (Table 1). In order to assess if different expression levels of *BRD4* were present in these different cell lines qPCR experiments in the GC cell lines AGP-01, ACP-02, ACP-03 originating from patients in Brazil, the Asian GC lines SNU-16 and KATO III, and HEK 293T cells were performed. No large differences were observed between the cell lines, but the ACP-02 cell line was shown to have the highest amount of *BRD4*, whereas KATO III had the lowest amount of *BRD4* (Figure 4A), thus expression of the target did not predict inhibitor sensitivity of the studied cell lines. Efficacy of BET inhibitors in cell proliferation has been linked to the transcriptional downregulation of *c-MYC* in several different cancer models [33–36]. In order to understand if modulation of *c-MYC* plays also a role in gastric cancer, *c-MYC* expression studies were performed on cells treated with (+)-JQ1 and PNZ5. The inhibitors (+)-JQ1 and PNZ5 did indeed reduce *c-MYC* expression in most of the cell lines, although the effect was not very pronounced in SNU-16, which is a cell line with known *c-MYC* amplification [37] (Figure 4B). Interestingly, there was also no downregulation of *c-MYC* mRNA levels in ACP-02, despite having high *BRD4* levels and responding to BET inhibitor treatment. Treatment with the 2 BET inhibitors did not have an effect on *c-MYC* protein level (data not shown). Furthermore, there is no correlation between *c-MYC* expression levels and *BRD4* levels. However, to our surprise we saw some differential response of *c-Myc* mRNA levels upon treatment with PNZ5 as compared to JQ1 although they bind to BRD4 with similar affinity (Supplementary Table 3).

**Table 1:** *In vitro* cytotoxic activity of (+)-JQ-1 and PNZ5

IC50 (μM)		
	(+)JQ-1	PNZ5
**AGP-01**		
**24h**	0.60 (0.43 – 0.83)	0.95 (0.73 – 1.22)
**48h**	0.21 (0.18 – 0.25)	0.47 (0.42 – 0.53)
**72h**	0.12 (0.11 – 0.13)	0.34 (0.32 – 0.36)
**ACP-02**		
**24h**	0.58 (0.41 – 0.82)	1.33 (0.99 – 1.78)
**48h**	0.56 (0.41 – 0.78)	0.85 (0.71 – 1.01)
**72h**	0.29 (0.20 – 0.41)	0.61 (0.46 – 0.82)
**ACP-03**		
**24h**	0.15 (0.15 – 0.47)	0.47 (0.37 – 0.60)
**48h**	0.12 (0.11 – 0.13)	0.29 (0.26 – 0.32)
**72h**	0.09 (0.08 – 0.10)	0.22 (0.20 – 0.24)
**Kato III**		
**24h**	>5	>5
**48h**	>5	>5
**72h**	>5	>5
**SNU-16**		
**24h**	>5	>5
**48h**	>5	>5
**72h**	0.66 (0.41 – 1.07)	1.34 (0.93 - 1.93)

Five cell lines were exposed to (+)-JQ1 and DDX (0.01 − 50 μM). IC_50_ values (μM) and confidence interval of 95% obtained by the Resazurin assay in gastric cancer cell lines after 24, 48 and 72 hours of exposure. Experiments were performed in triplicates and means from three independent experiments are reported.

### BET inhibitors display cytotoxicity and induce apoptosis in gastric cancer cell lines

PNZ5 and (+)-JQ1 are both potent and selective BET inhibitors, but belong to different chemical classes. We therefore performed a more detailed analysis of the antiproliferative effect of these 2 compounds in a high-content image screen (Howarth *et al.* unpublished data). (+)-JQ1 was the most potent compound in all cell lines (Table 1). Although at early time points there seemed to be small differences in the sensitivity of the cells treated with (+)-JQ1 or PNZ5, after 72 h there was no longer a statistical difference between the inhibitors. Based on our results, there was a tendency of the effect of PNZ5 to be time-dependent in all tested cell lines as the IC_50_ decreased over time, whereas (+)-JQ1 activity appeared to be time- and cell type-dependent (Table 1). The most sensitive cell line for both compounds was ACP-03, a primary intestinal-type adenocarcinoma and the most resistant was ACP-02, a diffuse-type adenocarcinoma cell line (Table 1). The compounds were then tested against 2 GC cell lines of Asian origin, which proved more resistant to BET inhibition. Growth of SNU-16 cells was only inhibited after 72h of treatment, with an IC_50_ of 0.66 and 1.34 μM for (+)-JQ1 and PNZ5 respectively, whereas proliferation of KATO III was unaffected by BET inhibition (Table 1).

**Figure 5:**
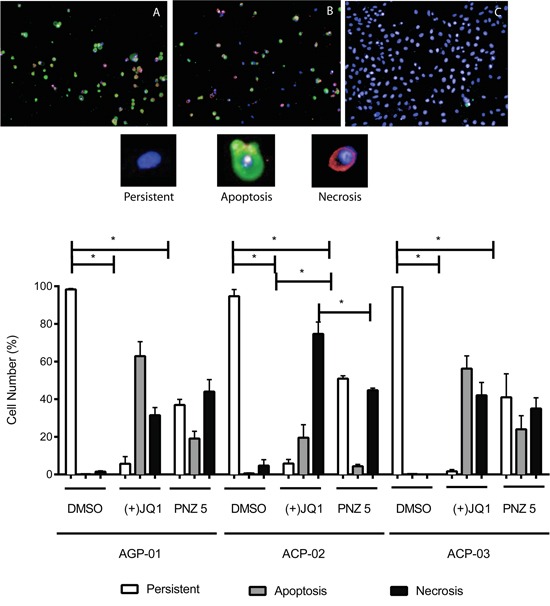
High content images of *in vitro* cytotoxic activity of (+)-JQ1 **A.** and PNZ5 **B.** on cell death pattern in gastric cancer cell lines compared to DMSO control **C.** (upper panel) Apoptosis increased after 72 hours exposure (lower panel). Apoptotic cells were defined as Annexin V positive with- or without Yo-Pro 3 uptake; Necrotic cells were defined as Yo-Pro 3 positive; and Persistent cells were defined as Annexin V and Yo-Pro 3 negative. *Hoechst* was used to identify the nuclei. Results are shown as mean +/− SEM from triplicates of two independent experiments.*p<0.01

In order to determine if the two compounds lead to the same cell death pattern, we performed a triple staining protocol using a high content imaging system (Figure 5). Cells treated with (+)-JQ1 and PNZ5 induced apoptosis in the three cell lines; AGP-01, ACP-02 and ACP-03 (Figure 5). After 72h, (+)-JQ1 (400 nM) induced apoptosis and necrosis in 62% and 31% of AGP-01cells, whereas PNZ5 (400 nM) induced 19% and 44% respectively. As for ACP-02, (+)-JQ1 induced 19% and 74% and PNZ5 induced 4% and 45% of apoptosis and necrosis, respectively. In ACP-03, (+)-JQ1 induced 56% and 42% and PNZ5 induced 24% and 35% of apoptosis and necrosis, respectively. There was no significant difference between the two compounds in inducing either apoptosis or necrosis apart from induction of necrosis in the ACP-02 cell line, consistent with their identical mode of action. As expected, there was a significant reduction in persistent cells in all cell lines when compared to the DMSO control.

### BET inhibitors display cytotoxicity in 3D multicellular spheroids

**Figure 6:**
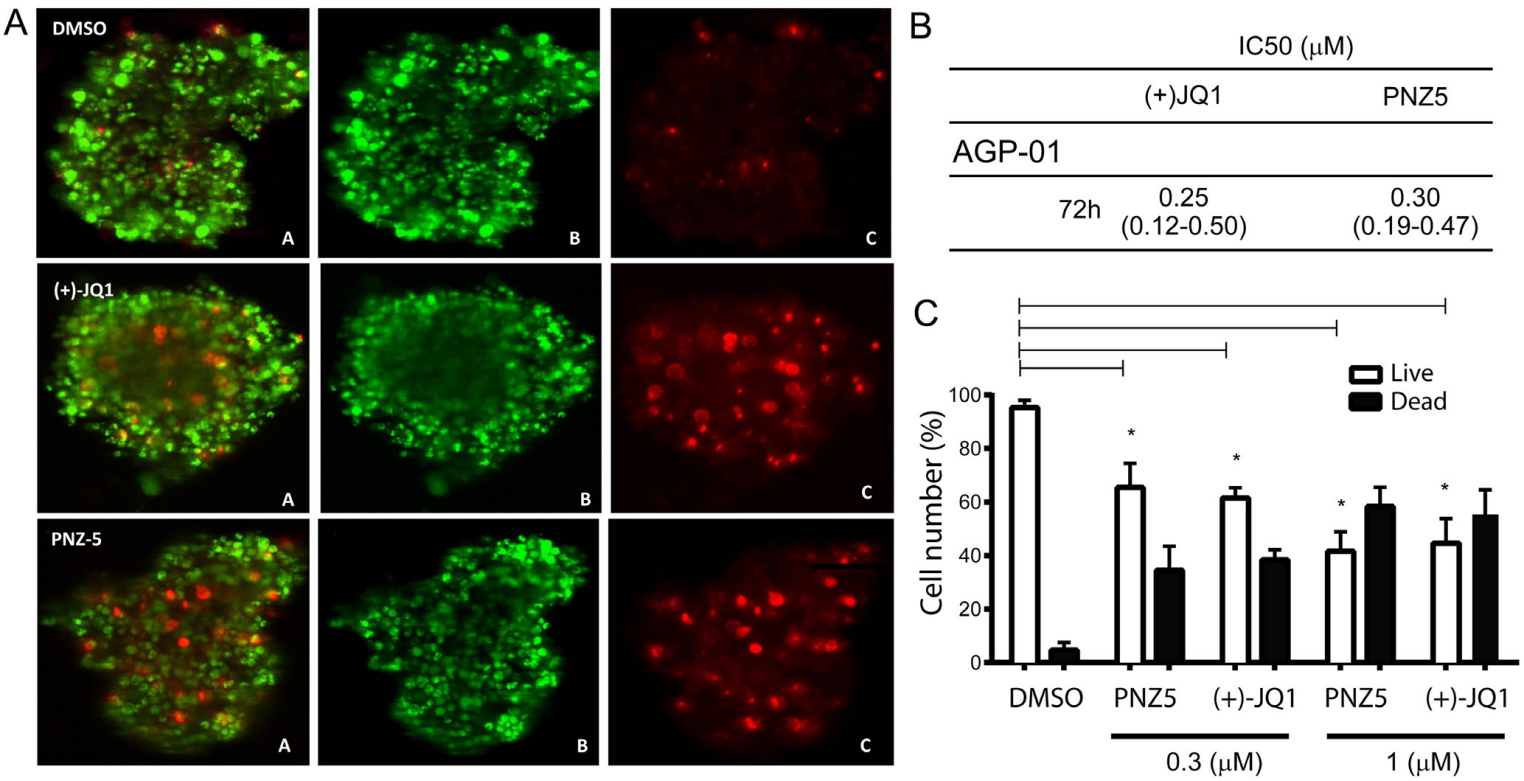
*In vitro* cytotoxic activity of (+)JQ-1 and PNZ5 on AGP-01 spheroids **A.** Live/Dead cell staining for AGP-01 spheroids treated with (+)JQ-1 (300nM) and PNZ-5 (300 nM). A. Merge; B. Calcein; C. EthD-1; **B.** IC_50_ values (μM) and confidence interval of 95% obtained by the Resazurin assay in AGP-01 spheroids 72 h of exposure; **C.** Live/Dead cell quantification. Experiments were performed in triplicates and means from three independent experiments are reported. *p<0.01

In addition to 2D cell monolayers, we also validated the cytotoxic effect of PNZ5 and (+)-JQ1 on a well-accepted three-dimensional (3D) *in vitro* model of cancer multicellular spheroids formed from AGP-01 cells. With the increase of drug dosage from 0.01μM to 1μM, the cells in the spheroids showed higher cell death, indicating an effective drug penetration and cytotoxicity in the 3D culture, which mimic the *in vivo* tumor tissue (Figure 6A and 6C).

The IC_50_ of PNZ5 and (+)-JQ1 in multicellular spheroids formed by AGP-01 cells were estimated to be 0.3 μM and 0.2 μM, respectively (Figure 6B) after 72 h of treatment. The IC_50_ values obtained from 3D multicellular spheroids were higher than the values from 2D monolayers of cell culture, however they were not statistically different, which implied that the two compounds are efficient in both 2D and 3D cell culture models.

## DISCUSSION

Herein, we describe inhibition of BET family members as a new possible strategy for the treatment/management of gastric cancer. Despite an overall decrease in incidence over the past decades, gastric cancer is still associated with poor survival. This is mainly due to the delayed signs and symptoms, such that at the time of diagnosis, 35% of GC patients have signs of metastases, 31% with peritoneal disease, 14% with liver metastases, and 16% with lung metastases [8]. Treatment of GC remains a major clinical challenge, especially for patients with metastasis. There is an urgent need for adjuvant and neoadjuvant trials evaluating novel targeted therapies in gastric cancer treatment.

In this study, BET inhibitors of two different scaffolds showed strong antiproliferative effects on three GC cell lines. PNZ5 is a newly identified BET isoxazole-based inhibitor with high selectivity and potency similar to the well-established (+)-JQ1, which is one of a range of diazepines that have been identified as potent BET ligands. A second class of key BET inhibitors are focused around a 3,5-dimethylisoxazole moiety, which was identified as an alternative acetyl-lysine mimic to replicate the same hydrogen-bond to a conserved arginine residue as does the native ligand. The most notable example to date is I-BET151, a highly potent and specific BET inhibitor, which has the advantage of improved pharmacokinetic properties compared to the triazolobenzodiazepine scaffolds [38–40]. PNZ5 is another example of a potent and selective isoxazole-based BET inhibitor with improved ligand efficacy. Interestingly, we observed some differences in the *c-MYC* expression levels when cells were treated with JQ1 or PNZ5. This effect was not expected and reasons for these variances are currently not well understood, but there might be differences in affinities for the different members of the BET family between JQ1 and PNZ5, which may mediate differential activation of antiproliferative pathways.

BET proteins are key mediators in the process of transcription of many genes involved in proliferation and anti-apoptotic and cell cycle progression [41]. BET specific inhibitors have shown therapeutic potential in many different tumor models through multiple mechanisms, including down-regulation of c-MYC expression [42, 15, 22, 36, 38, 43, 44]. c-MYC is a transcriptional factor involved in cell cycle regulation and has been described as a key element of gastric carcinogenesis [45–47]. The cells used in this study have been described previously to present chromosome 8 trisomy - the chromosome where the *MYC* oncogene is located [48]. Furthermore, frequency of *MYC* gain in tumors of patients with advanced GC seems to be higher in the Brazilian population [48–53] than in Asia, where Deng *et al*. [54] demonstrated the existence of five distinct gastric cancer patient subgroups, defined by the signature genomic alterations (FGFR2 [9% of tumours], KRAS [9%], EGFR [8%], ERBB2 [7%] and MET [4%]); however MYC was not one of them. The data obtained here is in line with these findings as both tested BET inhibitors only had little if any effect in the Asian cell lines tested, but showed potent antiproliferative effects in the cells of Brazilian origin, pointing to a potential genetic and demographic factor of GC.

Clonal high amplification of *c-MYC* is less frequent in diffuse-type gastric adenocarcinoma (ACP-02) than in ACP-03 and AGP-01 cells, originated from intestinal-type gastric adenocarcinoma [49, 50, 55]. In our study, we show that ACP-03 and AGP-01 were more sensitive to PNZ5 and (+)-JQ1 when compared to the ACP-02 cell line. Also, it seems that (+)-JQ1 and PNZ5 induce more apoptosis in ACP-03 and AGP-01 cell lines as compared to ACP-02 cells, where necrosis is the predominant *modus* of cell death.

Furthermore, our study shows that (+)-JQ1 and PNZ5 exposure reduces the expression of *c-MYC* in AGP-01 and ACP-03 cells, but not in ACP-02 cells, reinforcing the presence of different pathways involved in intestinal-type and diffuse-type gastric carcinogenesis [56]. Interestingly, although *c-MYC* expression is downregulated in the Asian derived-cell line KATO III and to a lesser extent in SNU-16, a cell line with high *c-MYC* amplification, there was a complete lack of antiproliferative activity of (+)-JQ1 and PNZ5, indicating that the antiproliferative effect is independent of *c-MYC* levels, a finding that has also been reported for leukemia cell lines [57]. The observed differences in antiproliferative response to BET inhibition may result from different genetic and/or epigenetic make up of these cell lines from different ethnic origin. The observations in the Brazilian cell lines corroborate previous work by Wyce *et al*. (2013) who demonstrated that prostate cancer cells with high c-MYC protein expression are more sensitive to I-BET762 compared to cell lines with low c-MYC protein expression [58]. The positive effect of BET inhibitors might be due to *c-MYC* transcriptional suppression since AGP-01 and ACP-03 cell lines present high amplification of *MYC* as shown previously [59].

Furthermore, Fowler *et al*. [60] demonstrate that cells with BRD4 amplification are more sensitive to (+)-JQ1. However, our own data indicate that ACP-02, which has the highest *BRD4* and *c-MYC* levels are less sensitive to BET inhibition (Figure 4) and there seems to be little correlation between BRD4 levels and MYC regulation and antiproliferative effect in the Asian gastric cancer cell lines. MYC independent mechanisms of tumor cell killing by BET inhibitors have also been reported in other tumors like osteosarcoma cells [61]. Taken together, we have identified BET inhibition as an alternative avenue for the treatment/management of gastric cancer in a selective group of patients and/or histological type of gastric cancer. The mechanisms leading to the sensitivity of gastric cell lines to BET inhibition and suitable biomarkes for patient stratification remain however to be established.

## MATERIALS AND METHODS

### Synthesis of PNZ5

See supporting information (Scheme 1)

### Protein expression and purification

BRD4 and other bromodomains were cloned, expressed and purified as described previously [21].

### Thermal shift assays

The recombinant bromodomains at 2 μM were mixed with 10 μM inhibitors. The assays and data evaluation for melting temperatures were performed using a Real-Time PCR Mx3005p machine (Stratagene) and the protocols previously described [62]. Experiments profiling the final compound (PNZ5) were performed in quadruplicates.

### Isothermal titration calorimetry

Calorimetric experiments were performed on a VP-ITC micro-calorimeter (MicroCal™, LLC Northampton, MA). Protein solution was buffer exchanged by dialysis into buffer 20 mM Hepes pH 7.5, 150 mM NaCl, and 0.5 mM TCEP. All measurements were carried out at 293.15 K while stirring at 286 rpm. The micro syringe was loaded with a protein solution of 250 μM, the compound solution was prepared at 20 μM and 2mL for the cell. All injections were performed using an initial injection of 2 μl followed by 34 injections of 8 μl with a duration of 16 sec per injection and a spacing of 240 sec between injection. The data were analysed with the MicroCal ORIGIN software package employing a single binding site model. The first data point was excluded from the analysis. Thermodynamic parameters were calculated (Δ*G* = Δ*H* - *T*Δ*S* = −R*T*lnK_B_ where Δ*G*, Δ*H* and Δ*S* are the changes in free energy, enthalpy and entropy of binding, respectively).

### Crystallization

BRD4 construct (Uniprot identifier as BRD4_HUMAN O60884-1 fragment 44-168) was used for crystallographic studies in complex with the inhibitor. Aliquots of the purified protein were set up for crystallization using a mosquito^®^ crystallization robot (TTP Labtech). Coarse screens were typically setup onto Greiner 3-well plates using three different drop ratios of precipitant to protein per condition (200+100 nL, 150+150 nL and 100+200 nL). All crystallizations were carried out using the sitting drop vapour diffusion method at 277.15 K. Small rod co-crystals of BRD4(1) in complex with the inhibitor (4 mM final concentration) were obtained by mixing 150 nL of the protein (12.5 mg/ml) and 150 nL crystallization buffer (20% PEG3350, 0.2 M zinc acetate, 0.1 M imidazole pH 7.8). A complete dataset was collected for BRD4(1) co-crystals at Diamond Light Source (beamline I04) and processed to 1.85 Å.

### Data collection and structure solution

Complex crystal was cryo-protected using the well solution supplemented with additional 20% ethylene glycol and was flash frozen in liquid nitrogen. Data was collected at Diamond Light Source beamline I04 at a wavelength of 0.9795 Å. Indexing and integration was carried out using XDS [63] and scaling was performed with AIMLESS [64]. Initial phases were calculated by molecular replacement with PHASER [65] using the apo template structure 2OSS.pdb. Unique and initial solutions were improved in a total of 50 cycles of automated protein chain tracing starting from existing model and computed using ARP/wARP [66]. Further manual building with COOT [67] and refinement against maximum likelihood target using REFMAC5 [68]. Thermal motions were analysed using TLSMD and hydrogen atoms were included in late refinement cycles. GRADE [69] was used to generate compound coordinates and cif files. All model validations were carried out using MolProbity [70]. Data collection and refinement statistics are compiled in Supplementary Table 4 The models and structure factors have been deposited with PDB accession codes: 5FBX.pdb

### Ethics statement

All samples were derived with written informed consent and approval from the University Hospital (Belém, Pará, Brazil) ethical review boards (protocol number: 142004).

### Gastric cancer cells

Our group established and characterized cytogenetically three new GC cell lines obtained from primary gastric adenocarcinoma (ACP02, diffuse type and ACP03, intestinal type) and peritoneal carcinomatosis (AGP01, ascitic fluid) from a patient with primary tumor on stomach (Intestinal-type adenocarcinoma), each of which exhibited a composite karyotype with several clonal chromosome alterations similar to primary tumor from the stomach as described previously [48]. All cell lines were maintained in Dulbecco's Modified Eagle Medium (DMEM) medium supplemented with 10% fetal bovine serum (FBS), 2 mM glutamine at 37°C with 5% CO2. Asian ethnicity cell lines, SNU-16 cell line (CRL-5974™), derived from a metastatic ascites of the stomach and Kato III (HTB-103™), derived from a metastatic site from a stomach primary tumor, were obtain from ATCC collection and cultured according to ATCC protocols.

### Chemicals

A library of Epigenetic compounds (Supplementary Table 1) was dissolved in DMSO to a concentration of 50 mM and stored at −20°C as a master stock solution. DMEM, FBS, GLUTAMAX, Annexin V, Yo-Pro 3 and *Hoechst* 33342 stain were purchased from Life Technologies, UK. Resazurin as sodium salt was purchased from Sigma, UK.

### Cytotoxicity against cancer cell lines

Initially, the epigenetic library compounds were tested in three cancer cell lines, at a single concentration of 10 μM for 72 h. (+)-JQ1 and PNZ5 (0.01-50 μM) were tested for cytotoxic activity against a panel of six gastric cancer cell lines: ACP-02 (Diffuse-type adenocarcinoma), ACP-03 (Intestinal-type adenocarcinoma) and AGP-01 (Malignant ascites) from Brazil and Kato III (Gastric metastasis), SNU- 16 (Gastric Ascites) from Asia. Each compound was dissolved in DMSO and diluted with media to obtain a maximum concentration of 50 μM. Cells (300 cells/well) were plated in 384-well flat-bottomed plates and cultured for 24h. Cells were exposed to a serial dilution of the compounds in DMEM with 1% FBS, for an additional 72 h. After the incubation time, the plates were centrifuged (1500rpm/3min), the supernatant removed and resazurin (10ng/ml) was added for 2h at 37°C before measurement of fluorescence at Ex 579nm/Em 584nm on an Envision microplate spectrophotometer (PerkinElmer). The concentration of compounds resulting in 50% growth inhibition (IC_50_) was calculated for each cell line in GraphPad Prism 5.0.

### Spheroids formation and drug treatment

GC cells AGP-01 in exponential growth phase were chosen for the spheroid formation. Briefly, 100 μL cell suspensions with the seeding density 1×10^3^ cells/μL were added to each well of a 96-well plate with an ultra-low cell adherence coating surface (Corning-Costar, UK), and were cultured in high-glucose Dulbecco's Modified Eagle Medium (DMEM, Lonza, UK) supplemented with 10% (v/v) foetal bovine serum (FBS, Life Technologies, US) and 100 U/mL penicillin − 100 μg/mL streptomycin (PAA, US), in a humidified incubator at 37°C and in an atmosphere of 5% CO2 in air. The spheroids were imaged and monitored using an In Cell Analyser (GE, UK). Spheroids with stable structures and diameters had formed after three days. On the day commencing the treatment, to obtain the final concentration, drug was diluted into 100 μL solution at twice the desired dosage with DMEM without FBS before adding to the 100 μL media of the well, and incubated with the spheroids under standard culture condition for 72 h.

### Cell live/dead imaging and image process of spheroids

A viability/cytotoxicity kit (Invitrogen, UK) was used for imaging the live and dead cells. The red fluorescent ethidium homodimer-1 (EthD-1) only permeated through the cell membrane of the dead cells and stained their nuclei, while living cells allowed the penetration of non-fluorescent acetomethoxy derivative of calcein (calcein AM) and degraded it into the green fluorescent calcein. The spheroid in each well of the 96-well plates was visualised and processed using a high-throughput imaging system, the In Cell Analyser (GE, UK). To process the 3D images, In Cell Investigator software (GE, UK) was used. Briefly, a background threshold was set for all the fluorescent images. The segmentation was selected using an iterative process of erosions and dilations. This allowed us to calculate a cell death percentage for the whole spheroid, indicated by the segmented red fluorescent areas.

### Hoechst 33342/Yo-Pro 3/annexin triple staining and live cell death pattern analysis

Cells exposed to (+)-JQ1 and PNZ5 (10μM) for 72 h were stained with Hoechst 33342 (1μM), Yo-Pro 3 (1μM) and Annexin V (0.3 μL per well) for 1 h. Cellular fluorescence was measured using the Operetta^®^, a high content imaging system (PerkinElmer, USA) using the following setup parameters: Brightfield transmitted light at 50% for 15 ms; *Hoechst* 33342 was excited by 35 ms exposure; Ex 360-400 nm/Em 410-480 nm, Yo-Pro 3 by 35 ms exposure; Ex 560-580 nm/Em 650-760 nm and Annexin V (Alexa 488) by 45 ms exposure; Ex 460-490 nm/Em 500-550 nm. All the generated data were analyzed by Harmony software and three categories were distinguished: persistent cells, apoptosis and necrosis - calculated as percentage of each class for every concentration used.

### BRD4 and c-MYC expression level

*c-MYC* expression was performed on AGP-01, ACP-02, ACP-03, SNU-16 and Kato III cell lines treated with (+)JQ1 and PNZ5 (500 nM) for 6h and collected for RNA extractions. For *BRD4* expression levels, cells were grown to 70% confluence and collected for RNA extractions. Total RNA was prepared by using a Qiagen RNEasy kit according to the manufacturer's instructions. cDNA were prepared by using The High Capacity cDNA Reverse Transcription Kit according to the manufacturer's manual. For cell line samples, an appropriate dilution of cDNA and gene-specific primers were combined with FAST SYBR^®^ Green MasterMix (Applied Bioscience) and amplified in a Light Cycler^®^ 480 II (Roche). All qPCR reactions were performed in triplicates. Ct (threshold cycle number) and expression values with standard deviations were calculated. *RPLP0* (human ribosomal protein, large, P0) was used as a housekeeping gene. Primer sequences for real-time PCRs were as follows: *c-MYC forward*, 5′-GCTGCTTAGACGCTGGATTT-3′; *c-MYC reverse*, 5′-TAACGTTGAGGGGCATCG-3′; *BRD4 forward*, 5′-AGGCAAAAGGAAGAGGA-3′; *BRD4* reverse, 5′-CGATGCTTGAGTTGTGTT-3′; *RPLP0 forward, 5′*-AGCCCAGAACACTGGTC-3′; *RPLP0 reverse, 5′-ACTCAGGATTTCAATGGT-3′. BRD4, c-MYC* and *RPLP0* primers were used at 0.25 μM final and exhibited PCR efficiencies of over 90%. Real-time amplification was performed with initial denaturation at 95°C for 30 sec, followed by 40 cycles of two-step amplification (95°C for 3 s, 60°C for 30 s).

### Fluorescence recovery after photobleaching (FRAP) assay

FRAP studies were performed using U2OS cells (purchased from ATCC) expressing a full-length BRD4 protein with an *N*-terminal eGFP as previously described [32]. In brief, six hours post-transfection the medium was replaced and 24 hours later imaging took place. The inhibitors were incubated with the cells for one hour before imaging. Half times of fluorescence recovery (t_1/2_) were calculated from individual recovery curves of twenty cells per group. Results were analysed according to their means and standard errors in GraphPad Prism 5.0. Data obtained from different experiments are presented as mean ± SEM from at least three independent experiments in triplicate and evaluated by analysis of variance (ANOVA) followed by Tukey test using a significance level of 5%.

## SUPPLEMENTARY MATERIALS, SUPPLEMENTARY SCHEMES




