# Supplementary material for: BET inhibition as a new strategy for the treatment of gastric cancer
Source: Oncotarget. 2016 Jun 1;7(28):43997–4012. doi: 10.18632/oncotarget.9766 (PMC5190074; doi:10.18632/oncotarget.9766)
Supplement: Supplementary file 1 [file oncotarget-07-43997-s001.pdf]

BET inhibition as a new strategy for the treatment of gastric cancer

SUPPLEMENTARY MATERIALS

Supplementary Table S1: Cytotoxicity Heatmap of Epigenetic Library compounds (*) against 3 cancer cell lines after 72 hours of exposure

Chemical Probe ID	Selectivity Profile	Probe Type	AGP-01	ACP-02	ACP-03
Bromosporine	pan	pan-Bromodomain inhibitor			
JQ1	BRD2, BRD3, BRD4, BRDT	Bromodomain inhibitor			
PFI-1	BRD2, BRD3, BRD4, BRDT	Bromodomain inhibitor			
PNZ5	BRD2, BRD3, BRD4, BRDT	Bromodomain inhibitor			
GSK2801	BAZ2B, BAZ2A	Bromodomain inhibitor			
I-CBP112	CREBBP, EP300	Bromodomain inhibitor			
SGC-CBP30	CREBBP, EP300	Bromodomain inhibitor			
OF-1	BRPF1, BRPF2, BRPF3	Bromodomain inhibitor			
PFI-4	BRPF1B	Bromodomain inhibitor			
BAZ2-ICR	BAZ2A, BAZ2B	Bromodomain inhibitor			
PFI-3	SMARCA2, SMARCA4, PB1(5)	Bromodomain inhibitor			
IOX1	Pan 2-OG	Pan -2-OG inhibitor			
GSK-J4	JMJD3, UTX	Demethylase inhibitor			
GSK-LSD1	LSD1	Demethylase inhibitor			
KDOM25	JARID1B	Demethylase inhibitor			
UNC1999	EHZ2/1	Methyltransferase Inhibitor			
UNC0638	G9a/GLP	Methyltransferase Inhibitor			
PFI-2	SETD7	Methyltransferase Inhibitor			
IOX2	PHD2/ELGN-1	Prolyl hydroxylase inhibitor			

Three gastric cancer cell lines were exposed to Epigenetic compounds (10 μM). Growth Inhibition (GI) was measured by the Resazurin assay at 72h. GI percentage values were estimated and compounds activity was coded by Green (GI < 10μM, GI > 90%), Orange (5 μM > GI < 10 μM, 50% < GI < 90%) and Red (> 10 μM, GI < 50%). Experiments were performed in triplicates and means from two independent experiments are reported. (*) http://www.thesgc.org/chemical-probes/epigenetics

Supplementary Table S2: SAR as assessed by thermal shift assay for compounds screened against BRD4(1). The mean ΔT_m is reported with the standard error of measurement, and the number of measurements recorded reported in brackets. All compounds were tested at 10 µM

See Supplementary File 1

Supplementary Table S3: ITC data of the interaction of PNZ5 and JQ1 with the first bromodomain of BRD4 (BRD4(1))

Ligand	N	K_D (nM)	ΔH (kcal/mol)	TΔS (kcal/mol)	ΔG (kcal/mol)
PNZ5	0.999 ± 0.002	5.43 ± 0.34	-15.57 ± 0.54	-4.66	-10.90
JQ1*	1.00 ± 0.001	49.0 ± 2.4	-8.42 ± 0.019	1.22	-8.42

*data according Filippakopoulos et al. (21)

Supplementary Table S4: Crystallographic data collection and refinement statistics

See Supplementary File 1

Supplementary Table S5: Protein stability shift data for human bromodomains in the presence of PNZ5 at 10 µM compound concentration

See Supplementary File 1

SUPPLEMENTARY SCHEMES

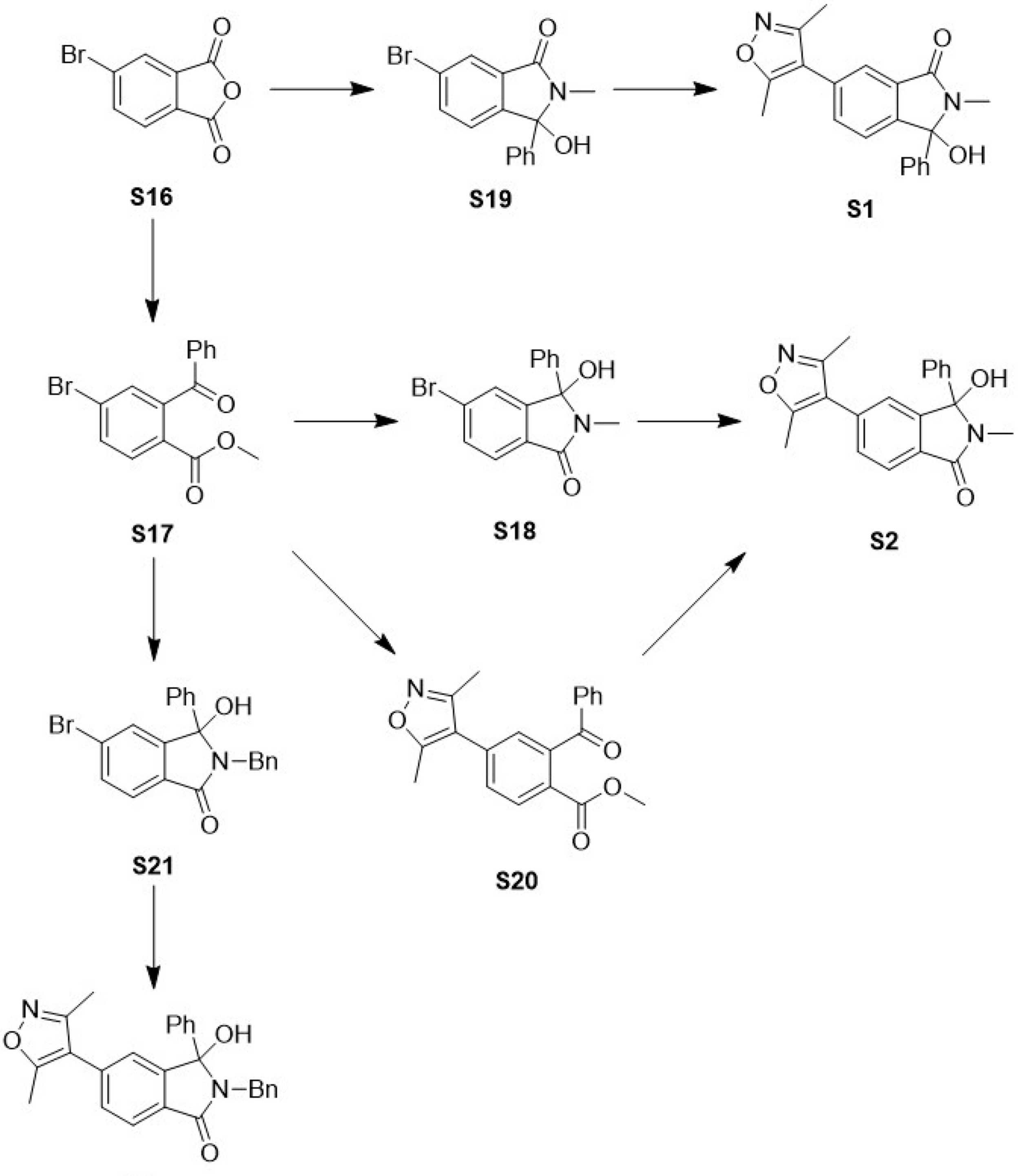

Scheme 1: Scaffold synthesis.

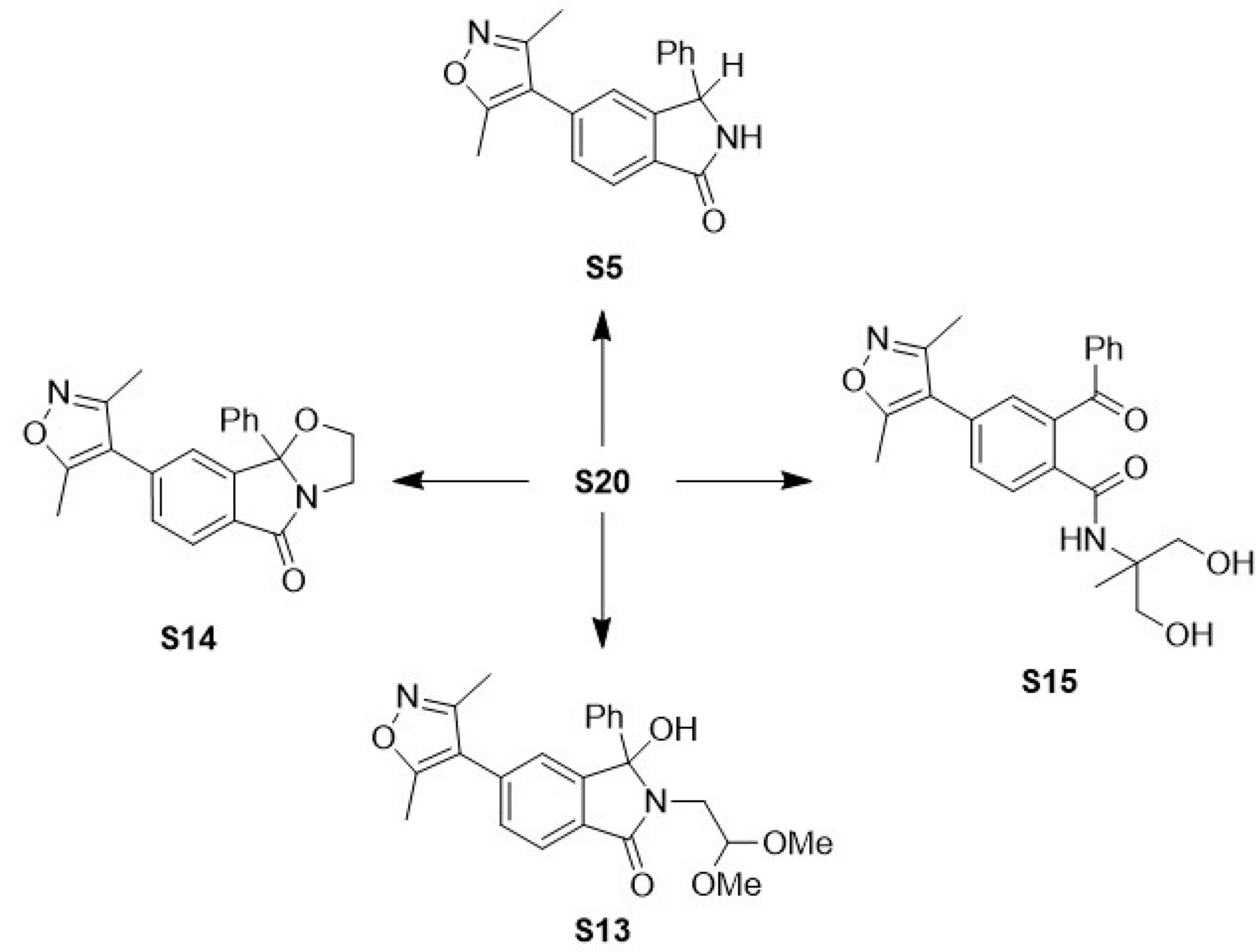

Scheme 2: ***N*-derivatisation.**

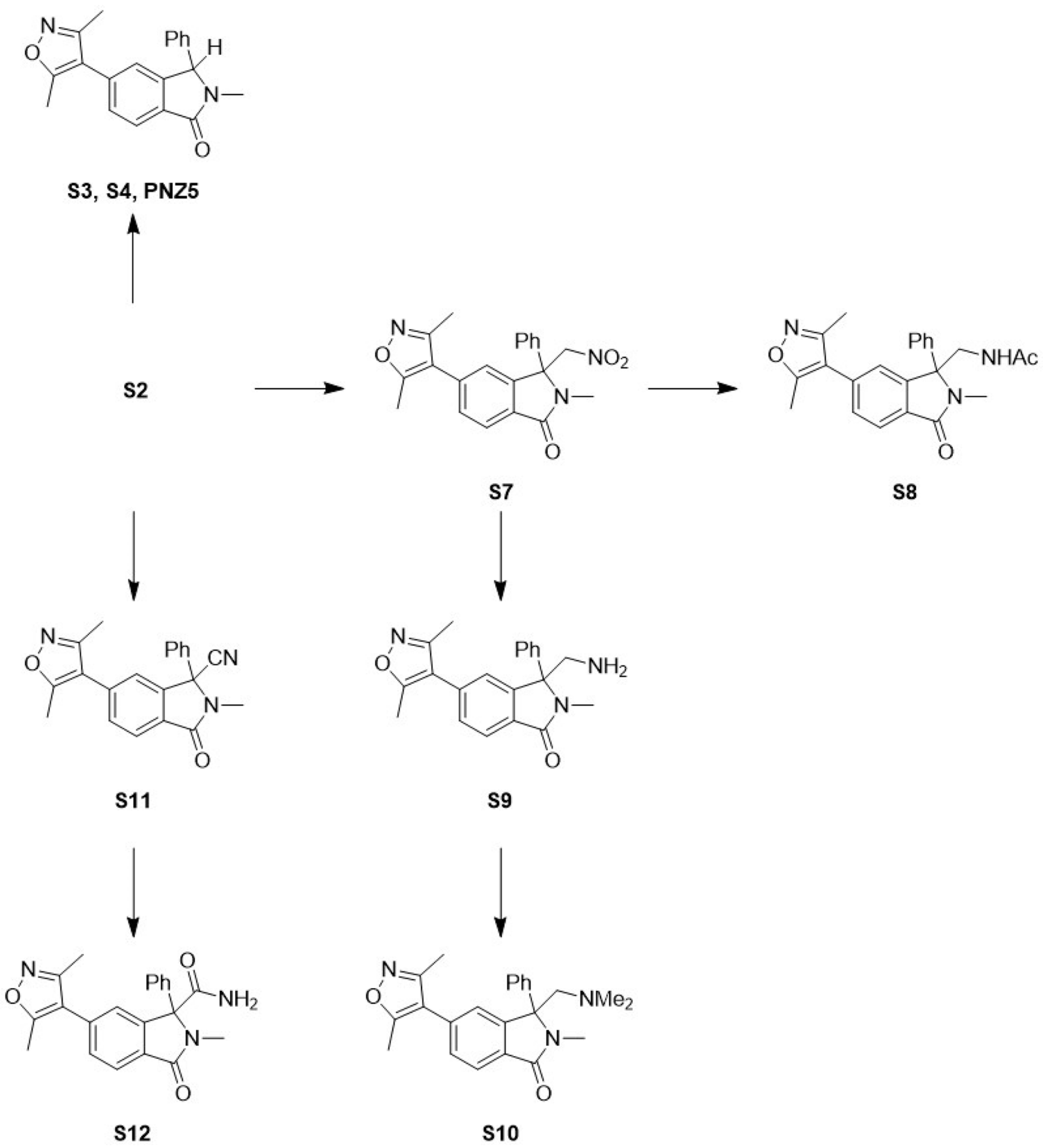

Scheme 3: Derivatisation of the hemiaminol.

General experimental details

Inert conditions are defined as reactions performed under argon (zero grade) in oven- and vacuum-dried glassware using standard vacuum line techniques and dry solvents, which have been passed through activated alumina columns. Degassing of solvents was achieved by bubbling through argon for 15 minutes with sonication. Reactions were monitored by thin layer chromatography using Merck silica gel 60 F254 plates and visualized by fluorescence quenching under UV light (254 nm) & by staining with potassium permanganate solution. Chromatographic purification was performed on VWR 60 silica gel 40-63 μM using technical grade solvents that were used as supplied. Reagents used were obtained from commercial suppliers or purified according to standard procedures with the following exceptions: Anhydrous 1,4-dioxane was dried over 3Å-molecular sieves.

NMR spectra were recorded on either a Bruker AVII400, a Bruker AVIII400 or a Bruker AVII 500, with ^{13}C cyroprobe, spectrometer. Chemical shifts (δ) are reported in ppm with the solvent resonance as the internal standard where appropriate. Data are reported as follows: multiplicity [s = singlet, d = doublet, t = triplet, q = quartet, quin = quintet, sxt = sextet, spt = septet, m = multiplet, br = broad], coupling constants (*J*) in hertz (Hz), integration, assignment. Two-dimensional spectroscopy (COSY, HSQC and HMBC) was performed to assist in structural elucidation but the data are not reported here. High-resolution mass spectra (ESI) were recorded on a Bruker Daltonics MicroTOF mass spectrometer. Infrared spectra were recorded on a Bruker Tensor 27 FT-IR spectrometer as a thin film, with only selected maximum absorbances reported in cm^{-1}. Optical rotations were recorded using a Perkin Elmer 341 polarimeter; absolute optical rotations are quoted as $[\alpha]_D^T$ where concentrations © are in g/100 mL, *D* refers to the D-line of sodium (589 nm), and temperatures (T) are given in degrees Celsius (°C). Melting points (Mpt) were obtained on a Leica Galen III Hot-stage melting point apparatus and microscope and on a Kofler hot block and are reported uncorrected. Enantiomers were resolved using an Agilent 1200 Series instrument using a semipreparative chiral stationary phase column.

Compound names were generated using the ACD/I-Lab service according to IUPAC nomenclature. Position numbering is for the purposes of assignment only and does not reflect IUPAC numbering conventions.

Abbreviations

ESI, electrospray ionisation; FCC, flash column chromatography; HPLC, high performance liquid chromatography; HR, high resolution; IR, infra-red; LR, low resolution; Mpt, melting point; MS, mass spectrometry; NMR, nuclear magnetic resonance; rt, room (ambient) temperature; v_{max}, maximum absorbance.

Synthetic methods

5-bromo-2-benzofuran-1,3-dione (S16)

NaOH (9.6 g, 240 mmol) was dissolved in H_2O (90 mL), phthalic anhydride (20 g, 120 mmol) was added and the reaction was stirred at rt until homogenous. The reaction was cooled to 0°C, Br_2 (6.8 mL, 132 mmol) was added dropwise and the reaction was stirred at 90°C for 6 h. The reaction was cooled at 3°C for 24 h, the resultant precipitate was filtered off, washed with H_2O (3 x 50 mL) and dried under vacuum. $SOCl_2$ (35 mL, 480 mmol) was added and the reaction was stirred at reflux for 2.5 h. The reaction mixture was filtered through celite, concentrated and recrystallised (EtOAc) to yield **S16** (14.6 g, 48%) as a pale yellow powder.

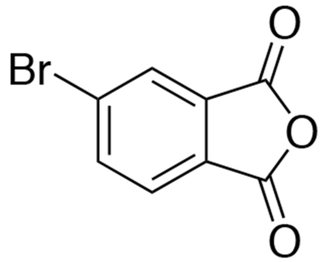

Mpt: 104-105°C (lit. 106-107°C); ^{1}H-NMR ($CDCl_3$): δ_H 8.17 (d, *J* = 1.9 Hz, 1H), 8.05 (dd, *J* = 8.1, 1.6 Hz, 1H), 7.90 (d, *J* = 8.1 Hz, 1H); ^{13}C-NMR ($CDCl_3$): δ_C 161.9, 161.6, 139.3, 132.9, 131.5, 129.8, 128.9, 126.8; LR-ESI-MS: $C_8H_3BrO_3Na$ $[M+Na]^+$ *m/z* found 248.9 calcd 248.9. Data are in accordance with literature values.[1]

Methyl 2-benzoyl-4-bromobenzoate (S17)

Under inert conditions, **S16** (34.7 g, 153 mmol) was dissolved in benzene (206 mL) and the solution was cooled to 0°C. $AlCl_3$ (42 g, 313 mmol) was added in portions and the reaction was stirred at rt for 24 h. The reaction mixture was poured into ice water (1 L) and extracted with EtOAc (3 x 500 mL). The combined organic extracts were dried over Na_2SO_4, filtered and concentrated. Separately $SOCl_2$ (28 mL, 383 mmol) was added dropwise to MeOH (115 mL) at 0°C. The original material was added to this solution and the reaction was stirred at rt for 14 h. The reaction mixture was concentrated and EtOAc (500 mL) was added. The organic phase was washed with H_2O (2 x 500 mL), dried over Na_2SO_4, filtered and concentrated. EtOAc (200 mL) was added, the

solution was passed through a plug of silica and the silica was washed with EtOAc (500 mL). The combined eluent was concentrated and recrystallisation (MeOH) yielded **S17** (21.7 g, 44%) as an amorphous white wax.

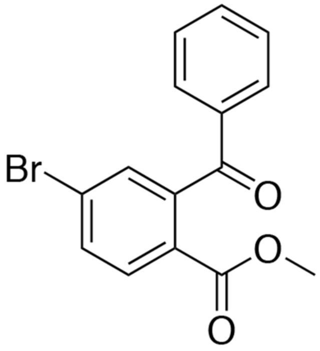

Mpt: 124.1-124.6°C (lit. 125°C); ^{1}H-NMR ($CDCl_3$): δ_H 7.93 (d, J = 8.5 Hz, 1H), 7.75 (dd, J = 8.2, 1.0 Hz, 2H), 7.72 (dd, J = 8.4, 2.0 Hz, 1H), 7.58 (tt, J = 7.3, 1.0 Hz, 1H), 7.55 (d, J = 2.2 Hz, 1H), 7.46 (t, J = 8.2 Hz, 2H), 3.62 (s, 3H); ^{13}C-NMR ($CDCl_3$): δ_C 195.2, 165.6, 143.3, 136.6, 133.4, 132.7, 131.6, 130.7, 129.2, 128.6, 127.9, 127.5, 52.3; LR-ESI-MS: $C_{15}H_{11}BrO_3Na$ $[M+Na]^+$ *m/z* found 341.0 calcd 341.0. Data are in accordance with literature values.[2]

5-bromo-3-hydroxy-2-methyl-3-phenylisoindolin-1-one (S18) and 6-bromo-3-hydroxy-2-methyl-3-phenylisoindolin-1-one (S19)

Method A. **S17** (470 mg, 1.6 mmol) was dissolved in $MeNH_{2(ethanolic)}$ (5.0 mL, 33 wt%) and the reaction was stirred at reflux for 14 h. The reaction mixture was concentrated and purification by FCC (20% EtOAc/pet. ether) yielded **S18** (320 mg, 68%) as a white solid.

Method B. NaOH (3.5 g, 87 mmol) was dissolved in H_2O (36 mL), phthalic anhydride (7.2 g, 43 mmol) was added and the solution was stirred until homogenous. The solution was cooled to 0°C, Br_2 (2.5 mL, 46 mmol) in H_2O (3.6 mL) was added dropwise and the reaction was stirred at 90°C for 6 h. The reaction mixture was cooled to 3°C for 24 h, the resultant precipitate was filtered, washed with H_2O (3 x 50 mL) and dried. $SOCl_2$ (14.5 mL) was added and the reaction was stirred at reflux for 2.5 h. The reaction mixture was concentrated, dissolved in EtOAc (200 mL), filtered and concentrated. Benzene (55 mL) and $AlCl_3$ (6.6 g, 49 mmol) were added and the reaction was stirred at rt for 12 h. The reaction mixture was poured over ice (200 mL) and the aqueous phase was extracted with EtOAc (4 x 200 mL). The combined organic extracts were washed with brine (100 mL), dried over Na_2SO_4, filtered and concentrated. Under inert conditions, the crude material was dissolved in tetrahydrofuran (120 mL), $SOCl_2$ (3.9 mL, 54 mmol) & a catalytic amount of *N,N*-dimethylformamide (15 drops) were added and the reaction was stirred at rt for 4 h. The reaction mixture was concentrated, $MeNH_{2(ethanolic)}$ (16 mL, 160 mmol, 33 wt%) was added and the reaction was stirred at rt for 14 h. The reaction mixture was concentrated and EtOAc (200 mL) was added. The organic phase was washed with H_2O (2 x 200 mL), brine (200 mL), dried over Na_2SO_4, filtered and concentrated. Purification by FCC (10% EtOAc/pet. ether) yielded **S18** (1.1 g, 6.9%) and **S19** (790 mg, 5.1%) as white solids. Recrystallisation of each sample (EtOAc) yielded **S18** (860 mg, 5.6%) and **S19** (470 mg, 3.1%) as white crystals.

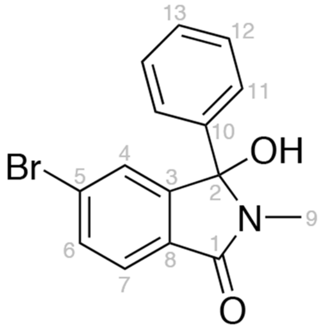

S18: Mpt: 190-192°C; ^{1}H-NMR ($CDCl_3$): δ_H 7.60 (d, J = 7.9 Hz, 1H, C(7)*H*), 7.58 (dd, J = 7.9, 1.6 Hz, 1H, C(6)*H*), 7.46 (d, J = 1.6 Hz, 1H, C(4)*H*), 7.41-7.35 (m, 5H, C(11)*H*, C(12)*H* & C(13)*H*), 2.80 (s, 3H, C(9)H_3); ^{13}C-NMR ($CDCl_3$): δ_C 166.6 (*C*(1)), 150.3 (*C*(3)), 137.0 (*C*(8)), 133.0 (*C*(6)), 129.2 (*C*(10)), 128.9 (*C*(11)), 128.8 (*C*(13)), 127.2 (*C*(5)), 126.2 (*C*(4)), 125.9 (*C*(12)), 124.7 (*C*(7)), 90.5 (*C*(2)), 24.1 (*C*(9)); ν_{max} 3209, 1680, 1429, 1065, 699; LR-ESI-MS: $C_{15}H_{12}BrNO_2Na$ $[M+Na]^+$ *m/z* found 340.0 calcd 340.0, $C_{15}H_{11}BrNO_2$ $[M-H]^-$ *m/z* found 316.0 calcd 316.0; HR-ESI-MS: $C_{15}H_{12}BrNO_2Na$ $[M+Na]^+$ *m/z* found 339.9941 calcd 339.9944 error = 0.7 ppm.

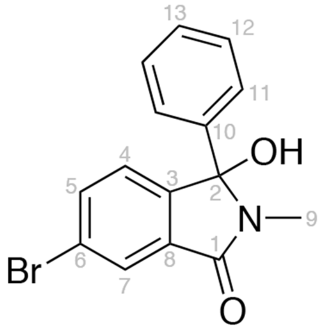

S19: Mpt: 170-171°C; ^{1}H-NMR ($CDCl_3$): δ_H 7.61 (dd, J = 8.0, 1.7 Hz, 1H, C(5)*H*), 7.50 (d, J = 1.6 Hz, 1H, C(7)*H*), 7.38-7.32 (m, 5H, C(11)*H*, C(12)*H* & C(13)*H*),

7.19 (d, J = 8.2 Hz, 1H, C(4)H), 2.71 (s, 3H, C(9)H_3); ^{13}C-NMR ($CDCl_3$): δ_C 166.6 (C(1)), 147.7 (C(3)), 137.3 (C(8)), 135.7 (C(5)), 131.7 (C(10)), 128.7 (C(11)), 128.7 (C(13)), 125.9 (C(7)), 125.8 (C(12)), 124.0 (C(4)), 123.5 (C(6)), 90.9 (C(2)), 24.1 (C(9)); ν_{max} 3238, 3068, 2925, 1688, 1443, 1085, 763; LR-ESI-MS: $C_{15}H_{12}BrNO_2Na$ $[M+Na]^+$ m/z found 340.0 calcd 340.0, $C_{15}H_{11}BrNO_2$ $[M-H]^-$ m/z found 316.0 calcd 316.0; HR-ESI-MS: $C_{15}H_{12}BrNO_2Na$ $[M+Na]^+$ m/z found 339.9944 calcd 339.9940 error = 0.9 ppm.

Methyl 2-benzoyl-4-(3,5-dimethyl-1,2-oxazol-4-yl) benzoate (S20)

In a pressure tube under inert conditions, **S17** (300 mg, 0.94 mmol), KOAc (190 mg, 1.9 mmol) and $PdCl_2$ (8.3 mg, 0.05 mmol) were combined. Separately 3,5-dimethylisoxazole (0.14 mL, 1.4 mmol) was added to *N,N*-dimethylacetamide (4.5 mL) under inert conditions and the solution was degased. This solution was added to the tube, the tube was sealed and the reaction was stirred at 130°C for 6 h. The reaction mixture was passed through a plug of celite and the celite was washed with CH_2Cl_2 (50 mL). The combined filtrate was concentrated and dissolved in EtOAc (150 mL). The organic phase was washed with H_2O (2 x 150 mL), dried over Na_2SO_4, filtered and concentrated. Purification by FCC (16% EtOAc/hexane) yielded **S20** (250 mg, 80%) as a pale yellow oil.

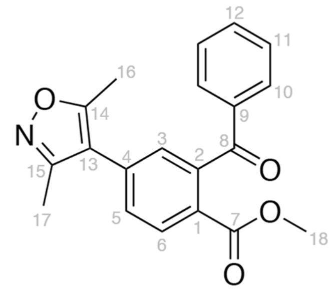

^{1}H-NMR ($CDCl_3$): δ_H 8.13 (d, J = 8.1 Hz, 1H, C(6)H), 7.81-7.76 (m, 2H, C(10)H), 7.59 (tt, J = 7.3, 1.3 Hz, 1H, C(12)H), 7.50-7.44 (m, 3H, C(5)H and C(11)H), 7.30 (d, J = 1.5 Hz, 1H, C(3)H), 3.64 (s, 3H, C(18)H_3), 2.45 (s, 3H, C(16)H_3), 2.31 (s, 3H, C(17)H_3); ^{13}C-NMR ($CDCl_3$): δ_C 196.4 (C(8)), 166.2 (C(14)), 165.9 (C(7)), 158.2 (C(15)), 142.4 (C(2)), 136.9 (C(9)), 135.1 (C(4)), 133.4 (C(12)), 130.7 (C(6)), 129.9 (C(5)), 129.2 (C(10)), 128.7 (C(11)), 128.1 (C(1)), 128.0 (C(3)), 115.2 (C(13)), 52.3 (C(18)), 11.8 (C(16)), 10.9 (C(17)); ν_{max} 3060, 2953, 2928, 1720, 1673, 1274, 1090, 708; LR-ESI-MS: $C_{20}H_{18}NO_4$ $[M+H]^+$ m/z found 336.1 calcd 336.1, $C_{20}H_{17}NO_4Na$ $[M+Na]^+$ m/z found 358.1 calcd 358.1; HR-ESI-MS: $C_{20}H_{17}NO_4Na$ $[M+Na]^+$ m/z found 358.1038 calcd 358.1050 error = 3.3 ppm.

6-(3,5-dimethyl-1,2-oxazol-4-yl)-3-hydroxy-2-methyl-3-phenylisoindolin-1-one (S1)

In a pressure tube under inert conditions, **S19** (50 mg, 0.16 mmol), KOAc (31 mg, 0.31 mmol) and $PdCl_2$ (1.4 mg, 0.0080 mmol) were combined. 3,5-dimethylisoxazole (23 µL, 0.24 mmol) was added to *N,N*-dimethylacetamide (0.75 mL) under inert conditions and the solution was degased. This solution was added to the tube, the tube was sealed and the reaction was stirred at 130°C for 6 h. The reaction mixture was passed through a plug of celite and the celite was washed with CH_2Cl_2 (20 mL). The combined filtrate was concentrated and EtOAc (50 mL) was added. The organic phase was washed with H_2O (2 x 50 mL), dried over Na_2SO_4, filtered and concentrated. Purification by FCC (25% EtOAc/pet. ether) yielded **S1** (33 mg, 62%) as a white solid.

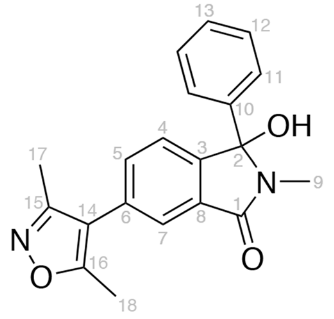

Mpt: 205-206°C; ^{1}H-NMR ($CDCl_3$): δ_H 7.64 (d, J = 0.9 Hz, 1H, C(7)H), 7.45-7.30 (m, 7H, C(4)H, C(5)H, C(11)H, C(12)H & C(13)H), 2.69 (s, 3H, C(9)H_3), 2.39 (s, 3H, C(18)H_3), 2.21 (s, 3H, C(17)H_3); ^{13}C-NMR ($CDCl_3$): δ_C 167.2 (C(1)), 165.9 (C(16)), 158.4 (C(15)), 148.0 (C(3)), 137.6 (C(8), 133.2 (C(5)), 132.0 (C(10)), 131.0 (C(6)), 128.8 (C(11)), 128.7 (C(13)), 125.9 (C(12)), 123.5 (C(7)), 123.3 (C(4)), 115.7 (C(14)), 91.0 (C(2)), 24.0 (C(9)), 11.6 (C(18)), 10.8 (C(17)); ν_{max} 3063, 2932, 1688, 1450, 1385, 1056, 765; LR-ESI-MS: $C_{20}H_{19}N_2O_3$ $[M+H]^+$ m/z found 335.1 calcd 335.1, $C_{20}H_{18}N_2O_3Na$ $[M+Na]^+$ m/z found 357.0 calcd 357.1, $C_{20}H_{17}N_2O_3$ $[M-H]^-$ m/z found 333.1 calcd 333.1; HR-ESI-MS: $C_{20}H_{18}N_2O_3Na$ $[M+Na]^+$ m/z found 357.1200 calcd 357.1210 error = 2.7 ppm.

5-(3,5-dimethyl-1,2-oxazol-4-yl)-3-hydroxy-2-methyl-3-phenylisoindolin-1-one (S2)

Method A. In a pressure tube under inert conditions, **S18** (1.28 g, 4.2 mmol), KOAc (0.83 g, 8.5 mmol) and $PdCl_2$ (38 mg, 0.21 mmol) were combined. 3,5-dimethylisoxazole (0.62 mL, 6.4 mmol) was added to *N,N*-dimethylacetamide (20 mL) under inert conditions and the solution was degased. This solution was added to the tube, the tube was sealed and the reaction was stirred at 130°C for 6 h. The reaction mixture was passed through

a plug of celite and the celite was washed with CH_2Cl_2 (50 mL). The combined filtrate was concentrated and EtOAc (250 mL) was added. The organic phase was washed with H_2O (2 x 250 mL), dried over Na_2SO_4, filtered and concentrated. Purification by FCC (33% EtOAc/pet. ether) yielded **S2** (1.17 g, 87%) as a white solid.

Method B. **S20** (120 mg, 0.36 mmol) was dissolved in $MeNH_{2(ethanolic)}$ (3 mL, 33 wt%) and the reaction was stirred at reflux for 16 h. The reaction mixture was concentrated and purification by FCC (50% EtOAc/pet. ether) yielded **S2** (29.8 mg, 25%) as a white solid.

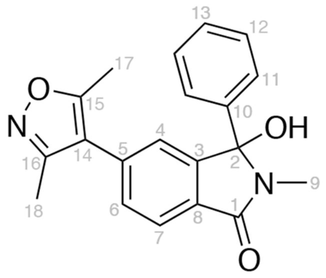

Mpt: 198-199°C; ^{1}H-NMR ($CDCl_3$): δ_H 7.66 (d, J = 7.8 Hz, 1H, C(7)*H*), 7.41-7.29 (m, 5H, C(11)*H*, C(12)*H* & C(13)*H*), 7.25 (dd, J = 7.8, 1.3 Hz, 1H, C(6)*H*), 7.19 (s, 1H, C(4)*H*), 5.02 (br s, 1H, O*H*), 2.64 (s, 3H, C(9)H_3), 2.33 (s, 3H, C(17)H_3), 2.16 (s, 3H, C(18)H_3); ^{13}C-NMR ($CDCl_3$): δ_C 167.4 (*C*(1)), 165.8 (*C*(15)), 158.2 (*C*(16))), 149.9 (*C*(3)), 137.7 (*C*(8)), 135.0 (*C*(5)), 129.9 (*C*(6)), 129.2 (*C*(10)), 128.7 (*C*(11) or *C*(12)), 128.6 (*C*(13)), 125.9 (*C*(11) or *C*(12)), 123.5 (*C*(7)), 123.3 (*C*(4)), 115.9 (*C*(14)), 91.0 (*C*(2)), 24.0 (*C*(9)), 11.6 (*C*(17)), 10.7 (*C*(18)); ν_{max} 2923, 2852, 1682, 1451, 1055, 699; LR-ESI-MS: $C_{20}H_{18}N_2O_3Na$ $[M+Na]^+$ *m/z* found 357.1 calcd 357.0, $C_{20}H_{17}N_2O_3$ $[M-H]^-$ *m/z* found 333.1 calcd 333.1; HR-ESI-MS: $C_{20}H_{18}N_2O_3Na$ $[M+Na]^+$ *m/z* found 357.1201 calcd 357.1210 error = 2.3 ppm.

5-(3,5-dimethyl-1,2-oxazol-4-yl)-2-methyl-3-(nitromethyl)-3-phenylisoindolin-1-one (S7)

Under inert conditions, **S2** (303 mg, 0.9 mmol) was dissolved in nitromethane (6.3 mL), diphenylphosphoric acid (45 mg, 0.18 mmol) was added and the reaction was stirred at reflux for 2 d. The reaction mixture was concentrated and EtOAc (150 mL) was added. The organic phase was washed with H_2O (2 x 150 mL), dried over Na_2SO_4, filtered and concentrated. Purification by FCC (1% MeOH/CH_2Cl_2) yielded **S7** (304 mg, 89%) as a colourless oil.

^{1}H-NMR ($CDCl_3$): δ_H 7.94 (d, J = 7.6 Hz, 1H, C(7)*H*), 7.44-7.36 (m, 4H, C(12)*H* & C(13)*H*), 7.19 (d, J = 0.6 Hz, 1H, C(4)*H*), 7.12-7.09 (m, 2H, C(6)*H* & C(14)*H*), 5.56 (d, J = 11.3 Hz, 1H, C(10)H_a), 5.34 (d, J = 11.3 Hz, 1H, C(10)H_b), 3.02 (s, 3H, C(9)H_3), 2.38 (s, 3H, C(18)H_3), 2.23 (s, 3H, C(19)H_3); ^{13}C-NMR ($CDCl_3$): δ_C 167.5 (*C*(1)), 165.9 (*C*(16)), 158.3 (*C*(17)), 146.0 (*C*(3)), 135.8 (*C*(8)), 134.9 (*C*(5)), 130.4 (*C*(14)), 130.2 (*C*(11)), 129.7 (*C*(12) or *C*(13)), 129.4 (*C*(6)), 125.4 (*C*(12) or *C*(13)), 124.5 (*C*(7)), 123.2 (*C*(4)), 115.9 (*C*(15)), 76.8 (*C*(10)), 69.0 (*C*(2)), 25.2 (*C*(9)), 11.6 (*C*(18)), 10.7 (*C*(19)); ν_{max} 3062, 2965, 2360, 1690, 1553, 1375, 699; LR-ESI-MS: $C_{21}H_{20}N_3O_4$ $[M+H]^+$ *m/z* found 378.1 calcd 378.1, $C_{21}H_{19}N_3O_4Na$ $[M+Na]^+$ *m/z* found 400.1 calcd 400.1; HR-ESI-MS: $C_{21}H_{19}N_3O_4Na$ $[M+Na]^+$ *m/z* found 400.1264 calcd 400.1268 error = 1.0 ppm.

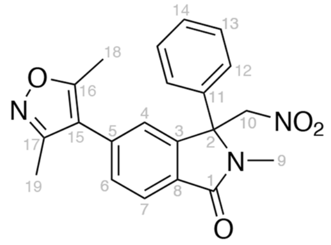

N-[(6-(3,5-dimethyl-1,2-oxazol-4-yl)-2-methyl-3-oxo-1-phenyl-2,3-dihydro-1*H*-isoindol-1-yl)methyl]acetamide (S8)

S7 (30 mg, 0.079 mmol) was suspended in AcOH (1 mL), the solution was cooled to 0°C, freshly ground zinc (52 mg, 0.79 mmol) was added in portions and the reaction was stirred at reflux for 14 h. The reaction mixture was concentrated and purification by FCC (1.5% MeOH/CH_2Cl_2) yielded **S8** (17 mg, 55%) as a white solid.

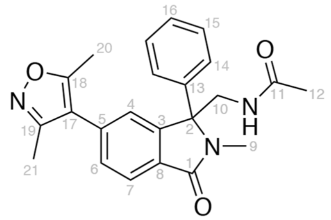

Mpt: 230.4-232.4°C; ^{1}H-NMR ($CDCl_3$): δ_H 7.85 (d, J = 7.3 Hz, 1H, C(7)*H*), 7.41-7.30 (m, 4H, C(6)*H*, C(15)*H* & C(16)*H*), 7.21 (d, J = 7.3 Hz, 2H, C(14)*H*), 7.15 (s, 1H, C(4)*H*), 6.03 (br s, 1H, N*H*), 4.99-4.90 (m, 1H, C(10)H_a), 3.96 (d, J = 13.2 Hz, 1H, C(10)H_b), 2.90 (s, 3H, C(9)H_3), 2.39 (s, 3H, C(20)H_3), 2.22 (s, 3H, C(21)H_3), 1.75 (s, 3H, C(12)H_3); ^{13}C-NMR ($CDCl_3$): δ_C 170.7 (*C*(11)), 169.2 (*C*(1)), 166.5 (*C*(18)), 158.8 (*C*(19)), 149.0 (*C*(3)), 137.5 (*C*(8)), 135.2 (*C*(5)), 131.0 (*C*(13)), 129.8 (*C*(6)), 129.8 (*C*(15)), 129.1 (*C*(16)), 126.4 (*C*(14)), 123.9 (*C*(7)), 123.7 (*C*(4)), 116.4 (*C*(17)), 71.1 (*C*(2)), 40.7 (*C*(10)), 25.7 (*C*(9)), 23.3 (*C*(12)), 12.1 (*C*(20)), 11.3 (*C*(21)); ν_{max} 3339, 3068, 2928, 1672, 697; LR-ESI-MS: $C_{23}H_{24}N_3O_3$ $[M+H]^+$ *m/z* found 390.2 calcd 390.1; HR-ESI-MS: $C_{23}H_{23}N_3O_3Na$ $[M+Na]^+$ *m/z* found 412.1632 calcd 412.1634 error = 0.6 ppm

3-(aminomethyl)-5-(3,5-dimethyl-1,2-oxazol-4-yl)-2-methyl-3-phenyl-2,3-dihydro-1*H*-isoindol-1-one (S9)

S7 (50 mg, 0.13 mmol) was dissolved in MeOH (4 mL), a solution of NH_4Cl (72.2 mg, 1.35 mmol) in H_2O (0.5 mL) & freshly-ground zinc powder (88.4 mg, 1.35 mmol) were added and the reaction was stirred at 60°C for 17 h. 1M HCl (10 mL) was added, the reaction mixture was filtered and reduced. H_2O (10 mL) was added and the aqueous phase was washed with EtOAc (3 x 20 mL). The aqueous phase was basified with 1M NaOH and extracted with EtOAc (3 x 20 mL). The combined organic extracts were dried over Na_2SO_4, filtered and concentrated to yield **S9** (39 mg, 85%) as a white powder.

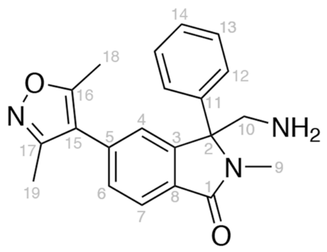

Mpt: 201.0-203.0°C; ^{1}H-NMR ($CDCl_3$): δ_H 7.95 (d, *J* = 7.9 Hz, 1H, C(7)*H*), 7.39-7.30 (m, 4H, C(6)*H*, C(13)*H* & C(14)*H*), 7.18 (d, *J* = 7.3 Hz, 2H, C(12)*H*), 7.09 (s, 1H, C(4)*H*), 3.85 (d, *J* = 13.9 Hz, 1H, C(10)H_a), 3.63 (d, *J* = 13.9 Hz, 1H, C(10)H_b), 2.89 (s, 3H, C(9)H_3), 2.34 (s, 3H, C(18)H_3), 2.19 (s, 3H, C(19)H_3); ^{13}C-NMR ($CDCl_3$): δ_C 168.8 (*C*(1)), 165.7 (*C*(16)), 158.2 (*C*(17)), 148.6 (*C*(3)), 137.7 (*C*(8)), 134.8 (*C*(5)), 131.2 (*C*(11)), 129.5 (*C*(6)), 129.2 (*C*(13)), 128.5 (*C*(14)), 126.1 (*C*(12)), 125.8 (*C*(7)), 124.1 (*C*(4)), 116.0 (*C*(15)), 71.4 (*C*(2)), 44.2 (*C*(10)), 25.1 (*C*(9)), 11.7 (*C*(18)), 10.8 (*C*(19)); ν_{max} 3394, 3330, 3060, 2927, 1693, 1378, 700; LR-ESI-MS: $C_{21}H_{22}N_3O_2$ $[M+H]^+$ *m/z* found 348.2 calcd 348.1; HR-ESI-MS: $C_{21}H_{22}N_3O_2$ $[M+H]^+$ *m/z* found 348.1707 calcd 348.1707 error = 0.2 ppm.

5-(3,5-dimethyl-1,2-oxazol-4-yl)-2-methyl-3-[(dimethylamino)methyl]-3-phenyl-2,3-dihydro-1*H*-isoindol-1-one (S10)

S9 (39 mg, 0.11 mmol) was suspended in H_2O (1 mL), formic acid (12 µL, 0.31 mmol) was added and the solution was cooled to 0°C. Formaldehyde (23 µL, 0.34 mmol) was added dropwise and the reaction was stirred at reflux for 7 h. The reaction mixture was basified with 1M NaOH and the aqueous phase was extracted with EtOAc (3 x 10 mL). The combined organic extracts were dried over Na_2SO_4, filtered and concentrated. Purification by FCC (0.5% MeOH/CH_2Cl_2) yielded **S10** (38 mg, 92%) as a white solid.

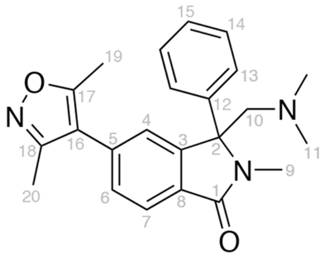

Mpt: 169.3-171.0°C; ^{1}H-NMR ($CDCl_3$): δ_H 8.00 (d, *J* = 7.6 Hz, 1H, C(7)*H*), 7.46-7.34 (m, 4H, C(6)*H*, C(14)*H* & C(15)*H*), 7.28 (dd, *J* = 8.0, 1.8 Hz, 2H, C(13)*H*), 7.14 (s, 3H, C(4)*H*), 3.55 (d, *J* = 13.7 Hz, 1H, C(10)H_a), 3.40 (d, *J* = 14.0 Hz, 1H, C(10)H_b), 2.99 (s, 3H, C(9)H_3), 2.41 (s, 3H, C(19)H_3), 2.26 (s, 3H, C(20)H_3), 2.11 (s, 6H, C(11)H_3); ^{13}C-NMR ($CDCl_3$): δ_C 168.7 (*C*(1)), 165.5 (*C*(17)), 158.3 (*C*(18)), 150.2 (*C*(3)), 139.1 (*C*(8)), 133.6 (*C*(5)), 131.1 (*C*(12)), 129.0 (*C*(14)), 128.9 (*C*(6)), 128.1 (*C*(15)), 125.8 (*C*(13)), 123.8 (*C*(7)), 122.7 (*C*(4)), 116.2 (*C*(16)), 70.4 (*C*(2)), 62.1 (*C*(10)), 47.6 (*C*(11)), 25.7 (*C*(9)), 11.6 (*C*(19)), 10.7 (*C*(20)); ν_{max} 2825, 2778, 1697, 1379, 701; LR-ESI-MS: $C_{23}H_{26}N_3O_2$ $[M+H]^+$ *m/z* found 376.2 calcd 376.2; HR-ESI-MS: $C_{23}H_{26}N_3O_2$ $[M+H]^+$ *m/z* found 376.2020 calcd 376.2016 error = 1.0 ppm.

6-(3,5)-dimethyl-1,2-oxazol-4-yl)-2-methyl-3-oxo-1-phenyl-2,3-dihydro-1*H*-isoindole-1-carbonitrile (S11)

Under inert conditions in a pressure tube, **S2** (330 mg, 0.99 mmol) was dissolved in tetrahydrofuran (7.3 mL), *t*-BuNC (131 µL, 1.14 mmol) & pTsOH·H_2O (19 mg, 0.099 mmol) were added, the tube was sealed and the reaction was stirred at 120°C for 15 h. EtOAc (50 mL) was added, the organic phase was washed with H_2O (3 x 50mL), dried over Na_2SO_4, filtered and concentrated. Purification by FCC (1.5% MeOH/CH_2Cl_2) yielded **S11** (254 mg, 75%) as a white powder.

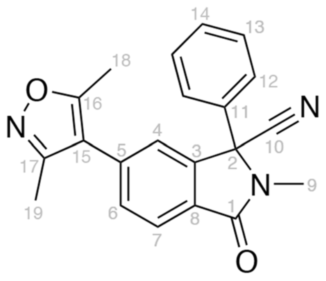

Mpt: 147.8-149.2°C; ^{1}H-NMR ($CDCl_3$): δ_H 8.01 (dd, *J* = 7.8, 0.5 Hz, 1H, C(7)*H*), 7.48 (dd, *J* = 7.8, 1.5 Hz, 1H, C(6)*H*), 7.47-7.43 (m, 3H, C(13)*H* & C(14)*H*), 7.43-7.34 (m, 2H, C(12)*H*), 7.21 (dd, *J* = 1.3, 0.6 Hz, 1H, C(4)*H*), 3.05 (s, 3H, C(9)H_3), 2.37 (s, 3H, C(18)H_3), 2.21 (s, 3H, C(19)H_3); ^{13}C-NMR ($CDCl_3$): δ_C 166.9 (*C*(1)), 166.1

(*C*(16)), 158.1 (*C*(17)), 144.6 (*C*(3)), 136.1 (*C*(8)), 133.1 (*C*(5)), 131.1 (*C*(6)), 130.3 (*C*(14)), 129.8 (*C*(13)), 129.0 (*C*(11)), 125.9 (*C*(12)), 124.8 (*C*(7)), 123.3 (*C*(4)), 116.0 (*C*(15)), 115.5 (*C*(10)), 66.3 (*C*(2)), 26.0 (*C*(9)), 11.7 (*C*(18)), 10.8 (*C*(19)); v_{max} 2918, 2322, 2207, 1703, 1359, 1011, 692; LR-ESI-MS: $C_{21}H_{18}N_3O_2$ $[M+H]^+$ *m/z* found 344.1 calcd 344.1; HR-ESI-MS: $C_{21}H_{17}N_3O_2Na$ $[M+Na]^+$ *m/z* found 366.1213 calcd 366.1209 error = 1.1 ppm.

6-(3,5-dimethyl-1,2-oxazol-4-yl)-2-methyl-3-oxo-1-phenyl-2,3-dihydro-1*H*-isoindole-1-carboxamide (S12)

S11 (375 mg, 1.09 mmol) was dissolved in CH_2Cl_2 (31 mL), concentrated H_2SO_4 (7.8 mL) was added and the reaction was stirred at reflux for 15 h. H_2O (35 mL) was added, the organic phase was separated and the aqueous phase was extracted with CH_2Cl_2 (3 x 35 mL). The combined organic phase was dried over Na_2SO_4, filtered and concentrated to yield **S12** (318 mg, 81%) as a white solid.

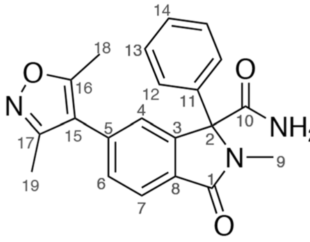

Mpt: >265°C; ^{1}H-NMR ($CDCl_3$): δ_H 7.95 (d, *J* = 7.9 Hz, 1H, C(7)*H*), 7.51 (d, *J* = 0.9 Hz, 1H, C(4)*H*), 7.46 (dd, *J* = 7.7, 1.4 Hz, 1H, C(6)*H*), 7.39-7.33 (m, 3H, C(13)*H* & C(14)*H*), 7.16-7.13 (m, 2H, C(12)*H*), 6.27 (br s, 1H, N*H*), 5.67 (br s, 1H, N*H*), 2.93 (s, 3H, C(9)H_3), 2.40 (s, 3H, C(18)H_3), 2.26 (s, 3H, C(19)H_3); ^{13}C-NMR ($CDCl_3$): δ_C 171.2 (*C*(10)), 168.7 (*C*(1)), 166.0 (*C*(16)), 158.2 (*C*(17)), 146.5 (*C*(3)), 136.0 (*C*(8)), 135.4 (*C*(5)), 130.1 (*C*(6)), 129.8 (*C*(11)), 129.0 (*C*(14)), 128.9 (*C*(13)), 128.3 (*C*(12)), 124.4 (*C*(4)), 124.1 (*C*(7)), 115.8 (*C*(15)), 75.7 (*C*(2)), 26.9 (*C*(9)), 11.8 (*C*(18)), 10.8 (*C*(19)); v_{max} 3351, 3156, 2924, 1682, 1374, 699; LR-ESI-MS: $C_{21}H_{20}N_3O_3$ $[M+H]^+$ *m/z* found 362.1 calcd 362.1; HR-ESI-MS: $C_{21}H_{19}N_3O_3Na$ $[M+Na]^+$ *m/z* found 384.1319 calcd 384.1308 error = 2.8 ppm.

5-(3,5-dimethyl-1,2-oxazol-4-yl)-2-methyl-3-phenyl-2,3-dihydro-1*H*-isoindol-1-one (S3), (*S*)-5-(3,5-dimethyl-1,2-oxazol-4-yl)-2-methyl-3-phenyl-2,3-dihydro-1*H*-isoindol-1-one (PNZ5) & ®-5-(3,5-dimethyl-1,2-oxazol-4-yl)-2-methyl-3-phenyl-2,3-dihydro-1*H*-isoindol-1-one (S4)

Method A. Under inert conditions, **S2** (200 mg, 0.60 mmol) was dissolved in CH_2Cl_2 (3.6 mL) and the solution was cooled to 0°C. Trifluoroacetic acid (0.6 mL) was added dropwise and the reaction was stirred at 0°C for 5 min. A solution of Et_3SiH (192 µL, 1.2 mmol) in CH_2Cl_2 (1.2 mL) was added slowly over 5 min and the reaction was stirred at rt for 4 h. The reaction mixture was concentrated, $NaHCO_{3(aq)}$ (20 mL) and CH_2Cl_2 (20 mL) were added and the organic phase was separated. The organic phase was washed with H_2O (2 x 20 mL), dried over Na_2SO_4, filtered and concentrated. Purification by FCC (1% MeOH/CH_2Cl_2) yielded **50** (64 mg, 34%) as a colourless oil.

Method B. **S2** (55 mg, 0.16 mmol) was dissolved in toluene (5 mL), diethyl 2,6-dimethyl-1,4-dihydropyridine-3,5-dicarboxylate (52 mg, 0.21 mmol) & diphenylphosphoric acid (2.0 mg, 0.008 mmol) were added and the reaction was stirred at rt for 7 d. EtOAc (20 mL) was added, the organic phase was washed with $NH_4Cl_{(aq)}$ (2 x 20 mL) & H_2O (2 x 20 mL), dried over Na_2SO_4, filtered and concentrated. Purification by FCC (1% MeOH/CH_2Cl_2) yielded **S3** (52 mg, 98%) as a colourless oil.

The enantiomers of **S3** were preparatively resolved by HPLC (stationary phase: Semipreparative Chiralpak AD, solvent composition: hexane:iso-propanol 70:30, flow rate: 1.15 $mL.min^{-1}$, injection concentration: 16.5 mg. mL^{-1}, injection volume: 100 µL, λ: 220 nm) with the enantiomers eluting at 43.9 min (**PNZ5**) and 55.2 min (**S4**).

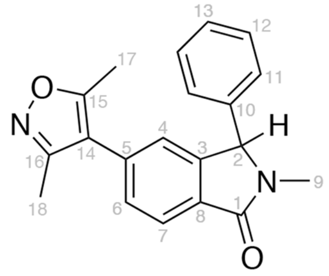

^{1}H-NMR ($CDCl_3$): δ_H 7.36 (dd, *J* = 7.7, 0.6 Hz, 1H, C(7)*H*), 7.40-7.29 (m, 5H, C(11)*H*, C(12)*H* & C(13)*H*), 7.24 (dd, *J* = 7.6, 1.5 Hz, 1H, C(6)*H*), 7.18 (s, 1H, C(4)*H*), 5.33 (s, 1H, C(2)*H*), 2.63 (s, 3H, C(9)H_3), 2.32 (s, 3H, C(17)H_3), 2.14 (s, 3H, C(18)H_3); ^{13}C-NMR ($CDCl_3$): δ_C 167.3 (*C*(1)), 165.8 (*C*(15)), 158.1 (*C*(16)), 149.9 (*C*(3)), 137.8 (*C*(8)), 134.9 (*C*(5)), 129.7 (*C*(6)), 129.2 (*C*(10)), 128.7 (*C*(12)), 128.6 (*C*(13)), 125.8 (*C*(11)), 123.4 (*C*(7)), 123.2 (*C*(4)), 115.9 (*C*(14)), 90.9 (*C*(2)), 23.9 (*C*(9)), 11.6 (*C*(17)), 10.6 (*C*(18)); v_{max} 3449, 2929, 2867, 1685, 1421, 702; LR-ESI-MS: $C_{20}H_{19}N_2O_2$ $[M+H]^+$ *m/z* found 319.1 calcd 319.2, $C_{20}H_{18}N_2O_2Na$ $[M+Na]^+$ *m/z* found 341.1 calcd 341.1; HR-ESI-MS: $C_{20}H_{19}N_2O_2$ $[M+H]^+$ *m/z* found 319.14410 calcd 319.14432 error = 0.67 ppm.

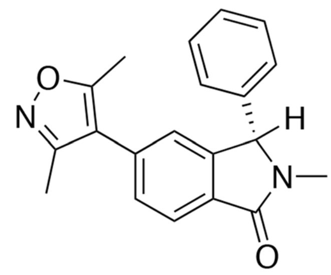

PNZ5: $[\alpha]_D^{20}$ - 25 (*c* 0.36, $CHCl_3$).

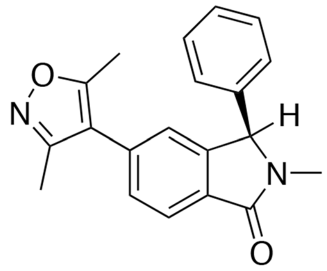

S4: $[\alpha]_D^{20}$ + 15 (*c* 0.21, $CHCl_3$).

2-(2,2-dimethoxyethyl)-5-(3,5-dimethyl-1,2-oxazol-4-yl)-3-hydroxy-3-phenyl-2,3-dihydro-1*H*-isoindol-1-one (S13)

S20 (103 mg, 0.31 mmol) was dissolved in EtOH (4 mL), aminoacetaldehyde dimethyl acetal (1 mL) and NEt_3 (0.1 mL) were added and the reaction was stirred at reflux for 14 h. The reaction mixture was concentrated and purification by iterative FCC (50% EtOAc/pet. ether then 5% MeOH/EtOAc) yielded **S13** (21 mg, 18%) as an opaque oil.

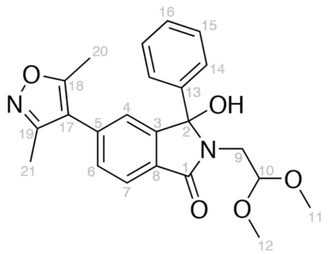

^{1}H-NMR ($CDCl_3$): δ_H 7.90 (dd, *J* = 7.7, 0.6 Hz, 1H, C(7)*H*), 7.42-7.32 (m, 6H, C(6)*H*, C(14)*H*, C(15)*H* & C(16)*H*), 7.13 (d, *J* = 0.8 Hz, 1H, C(4)*H*), 5.48 (br s, 1H, O*H*), 4.65 (dd, *J* = 6.9, 3.2 Hz, 1H, C(10)*H*), 4.07 (dd, *J* = 14.8, 3.2 Hz, 1H, C(9)H_a), 3.44 (s, 3H, C(11)H_3), 3.43 (s, 3H, C(12)H_3), 2.92 (dd, *J* = 14.8, 6.9 Hz, 1H, C(9)H_b), 2.34 (s, 3H, C(20)H_3), 2.20 (s, 3H, C(21)H_3); ^{13}C-NMR ($CDCl_3$): δ_C 167.9 (*C*(1)), 165.8 (*C*(18)), 158.3 (*C*(19)), 150.1 (*C*(3)), 139.0 (*C*(8)), 135.4 (*C*(5)), 129.9 (*C*(6)), 128.8 (*C*(15)), 128.7 (*C*(16)), 128.6 (*C*(16)), 126.1 (*C*(14)), 123.9 (*C*(7)), 123.0 (*C*(4)), 116.0 (*C*(17)), 102.3 (*C*(10)), 90.8 (*C*(2)), 55.4 (*C*(11)), 54.7 (*C*(12)), 41.6 (*C*(9)), 11.7 (*C*(20)), 10.8 (*C*(21)); ν_{max} 3317, 2935, 1703, 1626, 1409, 1124, 1059, 703; LR-ESI-MS: $C_{23}H_{25}N_2O_5$ $[M+H]^+$ *m/z* found 409.2 calcd 409.2; HR-ESI-MS: $C_{23}H_{24}N_2O_5Na$ $[M+Na]^+$ *m/z* found 431.15774 calcd 431.15786 error = 0.24 ppm.

8-(3,5-dimethyl-1,2-oxazol-4-yl)-9b-phenyl-2,3-dihydro[1,3]oxazolo[2,3-*a*]isoindol-5(9b*H*)-one (S14)

S20 (120 mg, 0.36 mmol) was dissolved in EtOH (3 mL), ethanolamine (1 mL) was added and the reaction was stirred at reflux for 14 h. EtOAc (20 mL) was added, the organic phase was washed with 1M HCl (3 x 20 mL), H_2O (3 x 20 mL), dried over Na_2SO_4, filtered and concentrated. Purification by iterative FCC (25% EtOAc/pet. ether then 1% MeOH/EtOAc) yielded **S14** (32 mg, 26%) as an opaque oil.

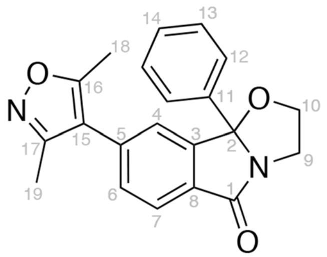

^{1}H-NMR ($CDCl_3$): δ_H 7.86 (dd, *J* = 7.8, 0.5 Hz, 1H, C(7)*H*), 7.61 (dd, *J* = 8.3, 1.5 Hz, 2H, C(12)*H*), 7.44-7.38 (m, 3H, C(13)*H* & C(14)*H*), 7.38 (dd, *J* = 7.8, 1.5 Hz, 1H, C(6)*H*), 7.15 (d, *J* = 0.7 Hz, 1H, C(4)*H*), 4.45-4.37 (m, 1H, C(10)H_a), 4.21-4.12 (m, 2H, C(10)H_b & C(9)H_a), 3.35-3.28 (m, 1H, C(9)H_b), 2.34 (s, 3H, C(18)H_3), 2.19 (s, 3H, C(19)H_3); ^{13}C-NMR ($CDCl_3$): δ_C 173.3 (*C*(1)), 165.9 (*C*(16)), 158.2 (*C*(17)), 147.5 (*C*(3)), 137.6 (*C*(8)), 135.8 (*C*(5)), 130.8 (*C*(6)), 130.2 (*C*(11)), 129.0 (*C*(14)), 128.8 (*C*(13)), 125.6 (*C*(12)), 124.7 (*C*(7)), 123.9 (*C*(4)), 115.8 (*C*(15)), 100.2 (*C*(2)), 70.2 (*C*(10)), 41.6 (*C*(9)), 11.6 (*C*(18)), 10.7 (*C*(19)); ν_{max} 3059, 2360, 2161, 1722, 1620, 1342, 1041, 702; LR-ESI-MS: $C_{21}H_{19}N_2O_3$ $[M+H]^+$ *m/z* found 347.1 calcd 347.1; HR-ESI-MS: $C_{21}H_{19}N_2O_3$ $[M+H]^+$ *m/z* found 347.13902 calcd 347.1392 error = 0.49 ppm.

2-benzoyl-*N*-(1,3-dihydroxy-2-methylpropan-2-yl)-4-(3,5-dimethyl-1,2-oxazol-4-yl)benzamide (S15)

S20 (101 mg, 0.30 mmol) was dissolved in EtOH (5 mL), 2-amino-2-methyl-1,3-propanediol (1.0 g, 9.5 mmol) & NEt_3 (0.1 mL) were added and the reaction was stirred at reflux for 2 d. The reaction mixture was concentrated

and purification by FCC (1% MeOH/CH_2Cl_2) yielded **S15** (52 mg, 42%) as an opaque oil.

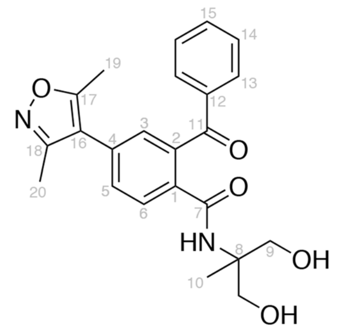

^{1}H-NMR ($CDCl_3$): δ_H 7.82 (dd, *J* = 8.3, 1.3 Hz, 2H, C(13)*H*), 7.72 (d, *J* = 7.8 Hz, 1H, C(6)*H*), 7.61 (tt, *J* = 7.6, 1.3 Hz, 1H, C(15)*H*), 7.47 (t, *J* = 7.8 Hz, 2H, C(14)*H*), 7.44 (dd, *J* = 7.8, 1.8 Hz, 1H, C(5)*H*), 7.33 (d, *J* = 1.8 Hz, 1H, C(3)*H*), 6.68 (br s, 1H, N*H*), 4.00 (br s, 2H, O*H*), 3.70 (d, *J* = 11.6 Hz, 2H, C(9)H_a), 3.58 (d, *J* = 11.6 Hz, 2H, C(9)H_b), 2.40 (s, 3H, C(19)H_3), 2.25 (s, 3H, C(20)H_3), 1.15 (s, 3H, C(10)H_3); ^{13}C-NMR ($CDCl_3$): δ_C 197.1 (*C*(11)), 168.9 (*C*(7)), 166.0 (*C*(17)), 158.2 (*C*(18)), 138.8 (*C*(2)), 136.5 (*C*(12)), 136.3 (*C*(4)), 133.8 (*C*(15)), 132.5 (*C*(1)), 131.0 (*C*(5)), 130.1 (*C*(14)), 129.6 (*C*(6)), 128.6 (*C*(13)), 127.9 (*C*(3)), 115.2 (*C*(16)), 67.5 (*C*(9)), 59.0 (*C*(8)), 19.0 (*C*(10)), 11.6 (*C*(19)), 10.7 (*C*(20)); ν_{max} 2916, 2358, 1702, 1417, 1059, 701; LR-ESI-MS: $C_{23}H_{25}N_2O_5$ $[M+H]^+$ *m/z* found 409.2 calcd 409.2, $C_{23}H_{24}N_2O_5Na$ $[M+Na]^+$ *m/z* found 431.2 calcd 431.2, $C_{23}H_{23}N_2O_5$ $[M-H]^-$ *m/z* found 407.1 calcd 407.2; HR-ESI-MS: $C_{23}H_{25}N_2O_5$ $[M+H]^+$ *m/z* found 409.17580 calcd 409.17659 error = 1.97 ppm.

5-(3,5-dimethyl-1,2-oxazol-4-yl)-3-phenyl-2,3-dihydro-1*H*-isoindol-1-one (S5)

S20 (62 mg, 0.15 mmol) was dissolved in EtOH (2 mL), allylamine (0.5 mL) was added and the reaction was stirred at reflux for 36 h. The reaction mixture was concentrated and purification by FCC (35% EtOAc/pet. ether) yielded **S5** (16 mg, 29%) as a white solid.

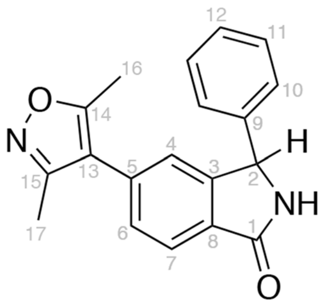

Mpt: 72.0-72.4°C; ^{1}H-NMR ($CDCl_3$): δ_H 7.91 (d, *J* = 7.8 Hz, 1H, C(7)*H*), 7.66 (br s, 1H, N*H*), 7.39-7.28 (m, 6H, C(6)*H*, C(10)*H*, C(11)*H* & C(12)*H*), 7.10 (s, 1H, C(4)*H*), 5.69 (s, 1H, C(2)*H*), 2.34 (s, 3H, C(16)H_3), 2.19 (s, 3H, C(17)H_3); ^{13}C-NMR ($CDCl_3$): δ_C 170.8 (*C*(1)), 165.7 (*C*(14)), 158.2 (*C*(15)), 148.6 (*C*(3)), 138.0 (*C*(8)), 134.7 (*C*(5)), 130.1 (*C*(9)), 129.2 (*C*(6)), 129.1 (*C*(11)), 128.6 (*C*(12)), 126.7 (*C*(10)), 124.1 (*C*(7)), 123.7 (*C*(4)), 116.0 (*C*(13)), 60.8 (*C*(2)), 11.6 (*C*(16)), 10.7 (*C*(17)); ν_{max} 3201, 3063, 2928, 1696, 1620, 730, 701; LR-ESI-MS: $C_{19}H_{17}N_2O_2$ $[M+H]^+$ *m/z* found 305.1 calcd 305.1; $C_{19}H_{16}N_2O_2Na$ $[M+Na]^+$ *m/z* found 327.1 calcd 327.1; HR-ESI-MS: $C_{19}H_{17}N_2O_2$ $[M+H]^+$ *m/z* found 305.12955 calcd 305.12860 error = 3.11 ppm.

2-benzyl-5-bromo-3-hydroxy-3-phenylisoindolin-1-one (S21)

S17 (200 mg, 0.63 mmol) was dissolved in EtOH (3.3 mL), benzylamine (2.0 mL) was added and the reaction was stirred at 60°C for 16 h. The reaction mixture was concentrated and purification by FCC (10% EtOAc/pet. ether) yielded **S21** (160 mg, 65%) as a white solid.

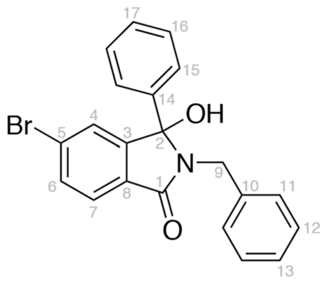

Mpt: 183-185°C; ^{1}H-NMR ($CDCl_3$): δ_H 7.64-7.46 (m, 2H, C(6)*H* & C(7)*H*), 7.36-7.06 (m, 11H, C(4)*H*, C(11)*H*, C(12)*H*, C(13)*H*, C(15)*H*, C(16)*H* & C(17)*H*), 4.61 (d, *J* = 14.9 Hz, 1H, C(9)H_a), 4.11 (d, *J* = 15.0 Hz, 1H, C(9)H_b); ^{13}C-NMR ($CDCl_3$): δ_C 167.1 (*C*(1)), 150.7 (*C*(3)), 137.6 (*C*(8)), 137.4 (*C*(10)), 132.9 (*C*(6)), 129.0 (*C*(14)), 128.8 (*C*(16)), 128.7 (*C*(17)), 128.6 (*C*(11)), 128.2 (*C*(15)), 127.4 (*C*(5)), 127.1 (*C*(13)), 126.3 (*C*(12)), 126.2 (*C*(4)), 124.8 (*C*(7)), 91.2 (*C*(2)), 43.1 (*C*(9)); ν_{max} 3230, 3064, 2937, 1672, 1062, 695; LR-ESI-MS: $C_{21}H_{16}BrNO_2Na$ $[M+Na]^+$ *m/z* found 416.0 calcd 416.0, $C_{21}H_{15}BrNO_2$ $[M-H]^-$ *m/z* found 392.0 calcd 392.0; HR-ESI-MS: $C_{21}H_{16}BrNO_2Na$ $[M+Na]^+$ *m/z* found 416.0247 calcd 416.0257 error = 2.2 ppm.

2-benzyl-5-(3,5-dimethyl-1,2-oxazol-4-yl)-3-hydroxy-3-phenylisoindolin-1-one (S6)

In a pressure tube under inert conditions, **S21** (20 mg, 0.050 mmol), KOAc (10 mg, 0.10 mmol) and $PdCl_2$ (0.5 mg, 0.003 mmol) were combined. 3,5-dimethylisoxazole (7.6 µL, 0.077 mmol) was added to *N,N*-dimethylacetamide (0.25 mL) under inert conditions and the solution was degased. This solution was added to the tube, the tube was sealed and the reaction was stirred

at 130°C for 6 h. The reaction mixture was passed through a plug of celite and the celite was washed with CH_2Cl_2 (20 mL). The combined filtrate was concentrated and dissolved in EtOAc (50 mL). The organic phase was washed with H_2O (2 x 50 mL), dried over Na_2SO_4, filtered and concentrated. Purification by FCC (25% EtOAc/pet. ether) yielded **S6** (13 mg, 61%) as a white solid.

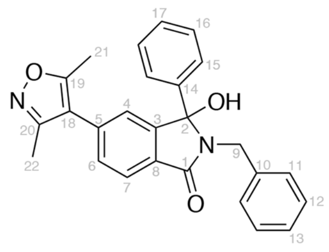

Mpt: 184-185°C; ^{1}H-NMR ($CDCl_3$): δ_H 7.88 (d, *J* = 7.9 Hz, 1H, C(7)*H*), 7.38-7.35 (m, 2H, C(12)*H*), 7.34 (dd, *J* = 7.9, 1.3 Hz, 1H, C(6)*H*), 7.32-7.28 (m, 3H, C(11)*H* & C(13)*H*), 7.25-7.22 (m, 2H, C(16)*H*), 7.20-7.14 (m, 3H, C(15)*H* & C(17)*H*), 7.13 (d, *J* = 0.9 Hz, 1H, C(4)*H*), 4.79 (d, *J* = 15.1 Hz, 1H, C(9)H_a), 4.10 (d, *J* = 15.1 Hz, 1H, C(9)H_b), 2.30 (s, 3H, C(21)H_3), 2.14 (s, 3H, C(22)H_3); ^{13}C-NMR ($CDCl_3$): δ_C 167.2 (*C*(1)), 166.8 (*C*(19)), 158.1 (*C*(20)), 149.8 (*C*(3)), 138.0 (*C*(8)), 137.9 (*C*(5)), 135.3 (*C*(14)), 130.2 (*C*(6)), 129.4 (*C*(10)), 128.7 (*C*(16)), 128.6 (*C*(13)), 128.6 (*C*(11)), 128.3 (*C*(15)), 127.2 (*C*(17)), 126.2 (*C*(12)), 123.9 (*C*(7)), 123.1 (*C*(4)), 115.9 (*C*(18)), 91.6 (*C*(2)), 43.1 (*C*(9)), 11.6 (*C*(21)), 10.7 (*C*(22)); ν_{max} 3063, 3032, 2925, 1700, 1409, 1055, 640; LR-ESI-MS: $C_{26}H_{22}N_2O_3Na$ $[M+Na]^+$ *m/z* found 433.1 calcd 433.2, $C_{26}H_{21}N_2O_3$ $[M-H]^-$ *m/z* found 409.1 calcd 409.2; HR-ESI-MS: $C_{26}H_{22}N_2O_3Na$ $[M+Na]^+$ *m/z* found 433.1517 calcd 433.1523 error = 1.2 ppm.

REFERENCES

1. Norman, M. H.; Kelley, J. L.; Hollingsworth, E. B. J. Med. Chem. 1993, 36, 3417–3423.
2. Unsinn, A.; Wunderlich, S. H.; Knochel, P. Adv. Synth. Catal. 2013, 355, 989–995.
3. Hay, D.; Fedorov, O.; Filippakopoulos, P.; Martin, S.; Philpott, M. Picaud S, Hewings DS, Uttakar S, Heightman TD, Conway SJ, Knapp S, Brennan PE. Med. Chem. Commun. 2012, 140–144
