# Supplementary material for: BET inhibition as a new strategy for the treatment of gastric cancer
Source: Oncotarget. 2016 Jun 1;7(28):43997–4012. doi: 10.18632/oncotarget.9766 (PMC5190074; doi:10.18632/oncotarget.9766)
Supplement: Supplementary file 2 [file oncotarget-07-43997-s002.docx]

**Supplemental Table S2:** SAR as assessed by thermal shift assay for compounds screened against BRD4(1). The mean ΔT­_m_ is reported with the standard error of measurement, and the number of measurements recorded reported in brackets. All compounds were tested at 10 µM.

| **Compound** | **Structure** | **ΔT_m_ Shift (^o^C)** |
| --- | --- | --- |
| **S2** |  | 8.23 ± 0.0 (1) |
| **S3** |  | 10.05 ± 0.10 (2) |
| **S4** |  | 3.58 ± 0.0 (1) |
| **PNZ5** |  | 11.36 ± 0.0 (1) |
| **S5** |  | 11.61 ± 0.0 (1) |
| **S6** |  | 7.17 ± 0.0 (1) |
| **S7** |  | 10.43 ± 0.77 (2) |
| **S8** |  | 4.47 ± 0.02 (2) |
| **S9** |  | 5.12 ± 0.02 (2) |
| **S10** |  | 6.53 ± 0.03 (2) |
| **S11** |  | 8.49 ± 0.11 (2) |
| **S12** |  | 7.19 ± 0.15 (2) |
| **S13** |  | 1.26 ± 0.0 (1) |
| **S14** |  | 8.25 ± 0.0 (1) |
| **S15** |  | 1.73 ± 0.0 (1) |

**Supplemental Table S4:** Crystallographic data collection and refinement statistics.

| PROTEIN ID | BRD4@1 |
| --- | --- |
| Ligand Structure |  |
|  |  |
| **Data collection** |  |
| Space Group | P 2_1_ 2_1_ 2_1_ |
| Cell Dimensions |  |
| a,b,c (Å) | 39.30 44.52 79.77 |
| α, β, γ (º) | 90.00 90.00 90.00 |
| Resolution (Å) | 29.71 (1.85)^a^ |
| Unique Observations | 12495 (735) |
| Completeness (%) | 99.6 (98.1) |
| Redundancy | 9.6 (9.7) |
| *R*_sym_^b^ or *R* _merge_ | 0.095 (0.998) |
| *I*/σ*I* | 18.9 (2.3) |
| Wavelength | 0.9795 |
| Phasing | MR |
|  |  |
| **Refinement** |  |
|  |  |
| R_work_^c^ / R_free_^d^ (%) | 21.2 / 26.6 |
| Number of atoms |  |
| protein / other / solvent | 1061 / 24 / 87 |
| B-Factors (Å^2^) |  |
| protein / other / solvent | 25.13 / 19.41 / 27.74 |
| R.M.S.D. Bond (Å) | 0.019 |
| R.M.S.D. Angle (^o^) | 2.006 |
| Ramachandran statistics |  |
| Allowed (%) | 100.00 |
| Favoured (%) | 98.41 |
| Outliers (%) | 0.00 |
|  |  |
| PDB ID | 5FBX.pdb |
|  |  |

^a^ Highest resolution outer shell (in Å) shown in parentheses.

^b^ R_sym_ = Σ|*I* − <*I*>|/Σ*I*

^c^ R_work_ = Σ||*F*obs| − |*F*calc||/Σ|*F*obs|, where *F*obs and *F*calc are the observed and calculated structure factors, respectively.

^d^ R_free_ was calculated with 5% of the data excluded from the refinement calculation.

MR = Molecular Replacement

**Supplementary Table 5:** Protein stability shift data for human bromodomains in the presence of PNZ5 at 10 μM compound concentration.

| **protein** | **Average ΔTm** | **StDev ΔTm** |
| --- | --- | --- |
| ASH1L | 0.41 | 0.23 |
| ATAD2 | 0.13 | 0.56 |
| BAZ1A | 1.20 | 1.14 |
| BAZ1B | 1.67 | 0.72 |
| BAZ2A | 0.32 | 0.20 |
| BAZ2B | 0.38 | 0.05 |
| BRD1 | 1.03 | 0.29 |
| BRD2(1) | 7.94 | 0.20 |
| BRD2(2) | 8.13 | 0.20 |
| BRD3(1) | 9.79 | 0.41 |
| BRD3(2) | 9.54 | 0.26 |
| BRD4(1) | 10.10 | 0.52 |
| BRD4(2) | 7.04 | 0.17 |
| BRD7 | 0.37 | 0.28 |
| BRD9 | 1.85 | 0.78 |
| BRDT(1) | 6.19 | 0.09 |
| BRDT(2) | 8.81 | 0.29 |
| BRPF1A | 0.81 | 0.26 |
| BRPF1B | 0.89 | 0.12 |
| BRPF3 | 0.95 | 0.25 |
| BRWD3(2) | 0.74 | 0.54 |
| CECR2 | 0.19 | 0.14 |
| CREBBP | 2.37 | 0.09 |
| EP300 | 2.49 | 0.10 |
| FALZ | 0.22 | 0.15 |
| GCN5L2 | 0.25 | 0.22 |
| ATAD2B | 0.30 | 0.32 |
| SP140L | 1.05 | 0.73 |
| MLL | 0.49 | 0.34 |
| PB1(1) | 0.79 | 0.38 |
| PB1(2) | 0.27 | 0.46 |
| PB1(3) | 0.12 | 0.28 |
| PB1(4) | 0.36 | 0.27 |
| PB1(5) | 0.28 | 0.23 |
| PB1(6) | 0.26 | 0.31 |
| PCAF | 0.45 | 0.24 |
| PHIP(2) | 0.22 | 0.59 |
| SMARCA2 | -0.18 | 0.35 |
| SMARCA4 | 0.04 | 0.18 |
| SP140 | 0.87 | 1.05 |
| TAF1(1) | 0.09 | 0.11 |
| TAF1(2) | 0.19 | 0.11 |
| TAF1L(1) | 0.31 | 0.21 |
| TAF1L(2) | 0.51 | 0.35 |
| TIF1-bromo | 0.86 | 0.48 |
| TIF1-phd-bromo | 0.06 | 0.18 |
| TRIM28 | -0.11 | 0.34 |
| WDR9(2) | 0.38 | 0.13 |
